# The kinetochore proteins CENP-E and CENP-F directly and specifically interact with distinct BUB mitotic checkpoint Ser/Thr kinases

**DOI:** 10.1074/jbc.RA118.003154

**Published:** 2018-05-10

**Authors:** Giuseppe Ciossani, Katharina Overlack, Arsen Petrovic, Pim J. Huis in 't Veld, Carolin Koerner, Sabine Wohlgemuth, Stefano Maffini, Andrea Musacchio

**Affiliations:** From the ‡ Department of Mechanistic Cell Biology, Max Planck Institute of Molecular Physiology, Otto-Hahn-Strasse 11, 44227 Dortmund and; the § Centre for Medical Biotechnology, Faculty of Biology, University Duisburg-Essen, Universitätsstrasse, 45141 Essen, Germany

**Keywords:** kinetochore, centromere, mitosis, checkpoint control, microtubule, Bub1, BubR1, CENP-E, CENP-F, spindle assembly checkpoint

## Abstract

The segregation of chromosomes during cell division relies on the function of the kinetochores, protein complexes that physically connect chromosomes with microtubules of the spindle. The metazoan proteins, centromere protein E (CENP-E) and CENP-F, are components of a fibrous layer of mitotic kinetochores named the corona. Several of their features suggest that CENP-E and CENP-F are paralogs: they are very large (comprising ∼2700 and 3200 residues, respectively), contain abundant predicted coiled-coil structures, are C-terminally prenylated, and are endowed with microtubule-binding sites at their termini. Moreover, CENP-E contains an ATP-hydrolyzing motor domain that promotes microtubule plus end–directed motion. Here, we show that both CENP-E and CENP-F are recruited to mitotic kinetochores independently of the main corona constituent, the Rod/Zwilch/ZW10 (RZZ) complex. We identified specific interactions of CENP-F and CENP-E with budding uninhibited by benzimidazole 1 (BUB1) and BUB1-related (BUBR1) mitotic checkpoint Ser/Thr kinases, respectively, paralogous proteins involved in mitotic checkpoint control and chromosome alignment. Whereas BUBR1 was dispensable for kinetochore localization of CENP-E, BUB1 was stringently required for CENP-F localization. Through biochemical reconstitution, we demonstrated that the CENP-E/BUBR1 and CENP-F/BUB1 interactions are direct and require similar determinants, a dimeric coiled-coil in CENP-E or CENP-F and a kinase domain in BUBR1 or BUB1. Our findings are consistent with the existence of structurally similar BUB1/CENP-F and BUBR1/CENP-E complexes, supporting the notion that CENP-E and CENP-F are evolutionarily related.

## Introduction

The segregation of chromosomes from a mother cell to its daughters during cell division relies on the function of specialized protein complexes, the kinetochores, as bridges linking chromosomes to spindle microtubules (1). Kinetochores are built on specialized chromosome loci known as centromeres, whose hallmark is the enrichment of the histone H3 variant centromeric protein A (CENP^3^-A, also known as CenH3) (2). CENP-A seeds kinetochore assembly by recruiting CENP-C, CENP-N, and their associated protein subunits in the constitutive centromere–associated network (3). These centromere proximal “inner kinetochore” subunits, in turn, recruit the centromere distal “outer kinetochore” subunits of the KMN complex (Knl1 complex, Mis12 complex, Ndc80 complex), which promote “end-on”' microtubule binding and control the spindle assembly checkpoint (SAC) (1).

**Figure 1.**
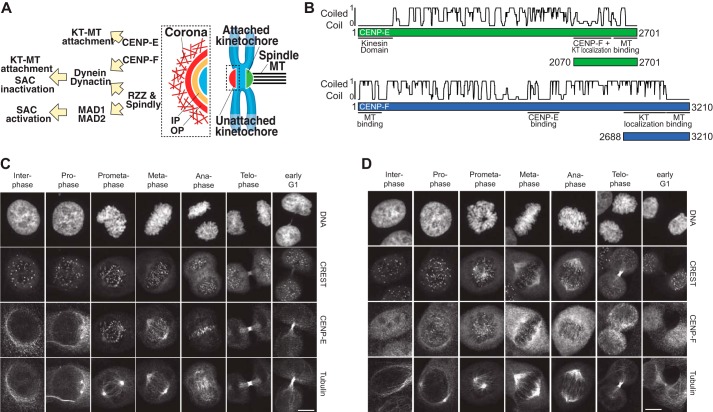
**Corona proteins CENP-E and CENP-F localize at kinetochores with distinct timing.** *A,* schematic representation of the corona structure and function. *MT,* microtubules; *IP,* inner plate of kinetochore; *OP,* outer plate of kinetochore. *B,* schematic organization of the CENP-E and CENP-F full-length proteins. Coiled-coil regions were predicted with programs Coils, Pcoils, and Marcoils (100–102) using default parameters (ncoils and paircoils, windows size 21). To combine all three coiled-coil prediction algorithms, we applied a scoring system in which we assigned for each residue two points for a high significance (*p* value > = 0.9) and one point for low significance (*p* value > = 0.8). Two additional points were granted for an identical register position in coiled-coil if predicted by all three programs, resulting in a maximum score of 8. *C* and *D,* representative images of fixed HeLa cells treated for fluorescence staining with the indicated antibodies. The panel illustrates the localization of CENP-E (*C*) and CENP-F (*D*) in the different phases of the cell cycle. *Scale bar,* 10 μm.

Early in mitosis, prior to end-on microtubule attachment, an additional fibrous structure, the kinetochore corona, assembles as the outermost layer of the kinetochore (Fig. 1*A*) (4–7). The corona's main constituent is a trimeric protein complex named RZZ (from the name of the fruit fly genes *Rough Deal* (ROD), *Zwilch*, and *Zeste White 10* (ZW10)). The ROD subunit is structurally related to proteins that oligomerize near biological membranes to promote vesicular trafficking, including clathrin (8, 9), leading to hypothesize that corona assembly results from RZZ polymerization (8). The interaction of the RZZ complex with an adaptor subunit named Spindly, in turn, further recruits the microtubule minus end–directed motor cytoplasmic dynein and its binding partner dynactin to kinetochores, as well as the MAD1/MAD2 complex, which is crucially required for SAC signaling (10–31).

Corona assembly leads to a broad expansion of the microtubule-binding interface of kinetochores that may promote initial microtubule capture, congression toward the metaphase plate, and SAC signaling (6, 26, 27, 32–35). Differently from the mature end-on attachments, initial attachments of kinetochores engage the microtubule lattice and are therefore defined as lateral or side-on. CENP-E, a kinesin-7 family member, plays a crucial role at this stage. Its inhibition or depletion leads to severe and persistent chromosome alignment defects, with numerous chromosomes failing to congress toward the spindle equator and stationing near the spindle poles, causing chronic activation of the SAC (6, 35–42). Human CENP-E consists of 2701 residues (Fig. 1*B*) (43). Besides the globular N-terminal motor domain, the rest of the CENP-E sequence forms a flexible and highly elongated (∼230 nm) coiled-coil (44). The kinetochore-targeting domain of CENP-E encompasses residues 2126–2476 and is followed by a microtubule-binding region (Fig. 1*B*) (45, 46). The distribution of CENP-E in an outer kinetochore crescent shape similar to that of the RZZ supports the notion that CENP-E is part of the kinetochore corona, but its persistence at kinetochores after disappearance of the corona suggests a corona-independent localization mechanism (6, 32, 33, 43, 47, 48).

CENP-F (also known as mitosin, 3210 residues in humans) is also a kinetochore corona constituent during early mitosis that persists at kinetochores after corona shedding (49–55). Like CENP-E, CENP-F is also highly enriched in predicted coiled-coil domains (Fig. 1*B*) but lacks an N-terminal motor domain. Rather, it contains two highly basic microtubule-binding domains in the N-terminal 385 residues and in the C-terminal 187 residues (56–58). Similarly to CENP-E, the kinetochore recruitment domain of CENP-F is positioned in proximity of the C terminus (encompassing residues 2581–3210, the minimal domain tested for this function to date (51, 53, 55)). The apparent similarity of CENP-E and CENP-F extends to the fact that they are both post-translationally modified with a farnesyl prenol lipid chain (isoprenoid) on canonical motifs positioned in their C termini (59). These modifications contribute to kinetochore recruitment of CENP-E and CENP-F, albeit to extents that differ in various reports (55, 60–62).

Previous studies identified CENP-F and BUBR1 as binding partners of CENP-E (37, 45, 63). BUBR1 is a crucial constituent of the SAC, a molecular network required to prevent premature mitotic exit (anaphase) in cells retaining unattached or improperly attached kinetochores (64). BUBR1 is a subunit of the mitotic checkpoint complex (MCC), the SAC effector (65). Its structure is a constellation of domains and interaction motifs required to mediate binding to other SAC proteins and terminates in a kinase domain (64). It has been proposed that CENP-E stimulates BUBR1 activity and that microtubule capture silences it (63, 66). Later studies, however, identified BUBR1 as an inactive pseudokinase (67, 68), and therefore, the significance of CENP-E microtubule binding for the role of BUBR1 in the SAC remains unclear. Depletion or inactivation of CENP-E, however, is compatible with a robust mitotic arrest (36, 38).

A yeast two-hybrid (Y2H) interaction of CENP-F and BUB1 has also been reported but never validated experimentally (45). BUB1, a paralog of BUBR1, retained genuine kinase activity in humans, and it plays a function at the interface of mitotic checkpoint signaling and kinetochore microtubule attachment (67, 69). Suggesting that the interaction of BUB1 and CENP-F is functionally important, previous studies identified BUB1 as being essential for kinetochore recruitment of CENP-F (69–72).

In our previous studies, we characterized in molecular detail how sequence divergence impacted the protein interaction potential of the human BUB1 and BUBR1 paralogs (73, 74). We described a molecular mechanism that explains how BUB1, through an interaction with a phospho-amino acid adaptor named BUB3, can interact with kinetochores and promote the recruitment of BUBR1 via a pseudo-dimeric interface (73–75). In view of these previous studies, here we have dissected the molecular basis of the interactions of BUBR1 and BUB1 with CENP-E and CENP-F. We provide strong evidence for the sub-functionalization of these paralogous protein pairs.

## Results and discussion

### Independent kinetochore localization of CENP-E, CENP-F, and the RZZ

Using specific antibodies (see under “Experimental procedures”), we assessed the timing and specificity of kinetochore localization of CENP-E, CENP-F, Zwilch, and MAD1. CENP-E showed perinuclear localization until prometaphase, when it first appeared at kinetochores. It persisted there until metaphase and was then found at the spindle midzone after anaphase onset (Fig. 1*C*). This localization, which corresponds to previous descriptions (36, 43), is reminiscent of that of chromosome passenger proteins (76). CENP-F, however, localized to kinetochores already in prophase, where it was also temporarily visible at the nuclear envelope, and persisted there until anaphase, with progressive weakening and dispersion (Fig. 1*D*), as noted previously (49, 50, 77–79). Also Zwilch (a subunit of the RZZ complex) and MAD1 were already visible at kinetochores in prophase, but they became invisible at these structures upon achievement of metaphase (Fig. S1, *A* and *B*), in agreement with the notion that the corona becomes dissolved upon microtubule attachment (see Introduction).

**Figure 2.**
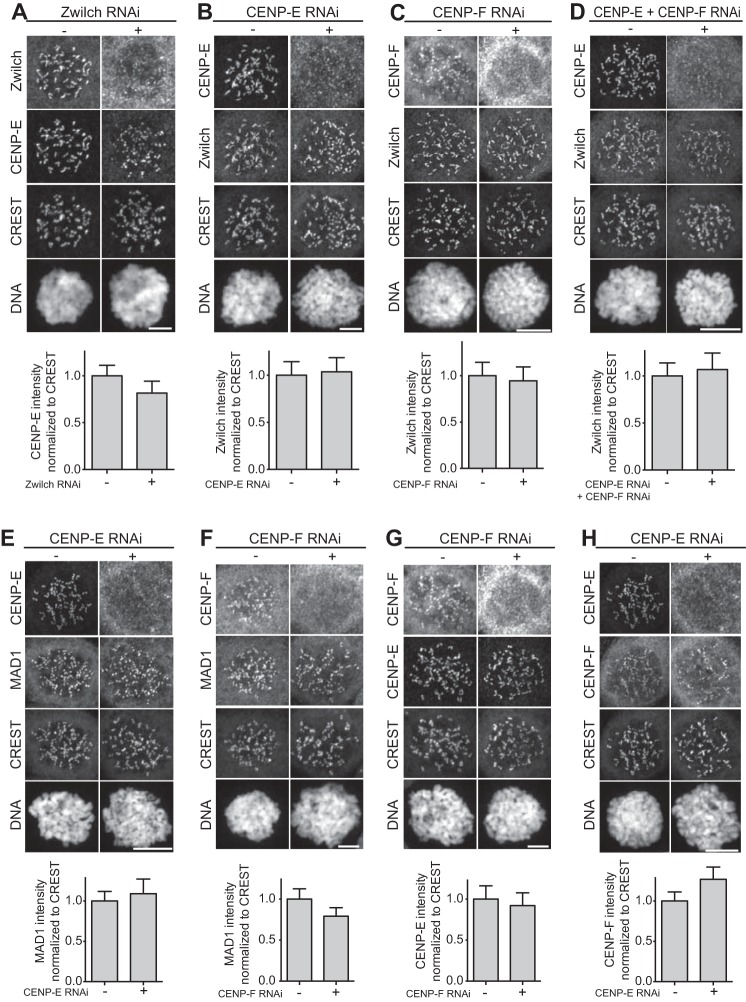
**Kinetochore localization of RZZ and MAD1 are independent of CENP-E and CENP-F.** *A–H,* representative images and quantification of protein kinetochore levels in HeLa cells mock-treated or depleted of Zwilch (*A*), CENP-E (*B, E,* and *H*), CENP-F (*C, F,* and *G*), or co-depleted of CENP-E and CENP-F (*D*). *Scale bar,* 10 μm. Zwilch depletion does not affect the localization of CENP-E (*A*). CENP-E depletion does not affect the localization of Zwilch (*B*), MAD1 (*E*), and CENP-F (*H*). Similarly, CENP-F depletion does not interfere with the recruitment of Zwilch (*C*), MAD1 (*F*), and CENP-E (*G*). Co-depletion of CENP-E and CENP-F has no effects on localization of Zwilch (*D*). The *graphs* show mean intensity of one (*B, C,* and *E*), two (*D* and *F*), or three (*A, G,* and *H*) experiments; the *error bars* indicate S.E., and the mean values for nondepleted cells are set to 1. Elements in the *left column* of *A* (negative controls of the RNAi experiments) are also shown in Fig. S3*C*. Elements in *G* are shown again in Fig. S2*E*.

Thus, both CENP-E and CENP-F continue to localize to kinetochores well beyond the timing of removal of the RZZ complex and MAD1, suggesting that they can be retained at kinetochores independent of the corona. To test this directly, we identified conditions for optimal depletion of Zwilch, CENP-E, or CENP-F by RNA interference (RNAi) (Fig. S2, *A–J*). Depletion of Zwilch resulted in depletion of MAD1 from kinetochores (Fig. S2, *H–J*) but left the kinetochore levels of CENP-E essentially untouched (Fig. 2*A*). This observation is in agreement with previous studies showing that MAD2, whose kinetochore localization requires MAD1 (80, 81), is also depleted from kinetochores upon depletion of other RZZ subunits (20, 28, 69). The observation that CENP-E retains kinetochore localization under conditions in which MAD1 appears to become depleted seems inconsistent with a recent report proposing that MAD1 is required for kinetochore localization of CENP-E (82) but agrees with previous reports that failed to detect consequences on CENP-E localization upon depletion of MAD1 (80, 81).

Conversely, depletion of CENP-E or CENP-F in HeLa cells, or even their co-depletion (Fig. S3*A*), did not influence the kinetochore localization of Zwilch (Fig. 2, *B–F*, and Fig. S4, *A–D*), as observed previously (81, 83). We also determined that CENP-E depletion did not have consequences for kinetochore localization of MAD1 (Fig. 2*E*). We note, however, that depletion of CENP-E in DLD-1 cells was reported to have deleterious effects on MAD2 localization (which requires MAD1), whereas MAD1 or RZZ subunits were not tested (72). Based on these results, we conclude that kinetochore localization of CENP-E and CENP-F does not require the kinetochore corona nor does it influence corona assembly. We also observed that CENP-E and CENP-F were not reciprocally affected by their depletion (Fig. 2, *G* and *H*), indicating that they localize (at least largely) independently to kinetochores, as suggested previously (37).

In most cells analyzed, depletion of CENP-F resulted in apparently normal metaphase alignment, with only a slight increase in the fraction of cells presenting metaphase alignment defects (Figs. S4*B* and S5). In agreement with the effects of CENP-F depletion being mild, duration of mitosis (caused by spindle assembly checkpoint activation) was only marginally increased in cells depleted of CENP-F (Fig. 2 and Fig. S5D). Similarly mild effects from depleting CENP-F were observed previously (56, 69, 83–85). In contrast, depletion of CENP-E (with or without additional depletion of CENP-F) led to conspicuous chromosome alignment problems (Fig. S4, *C* and *D*), as reported previously (6, 35–42).

### CENP-E binds to the BUBR1 pseudokinase domain

**Figure 3.**
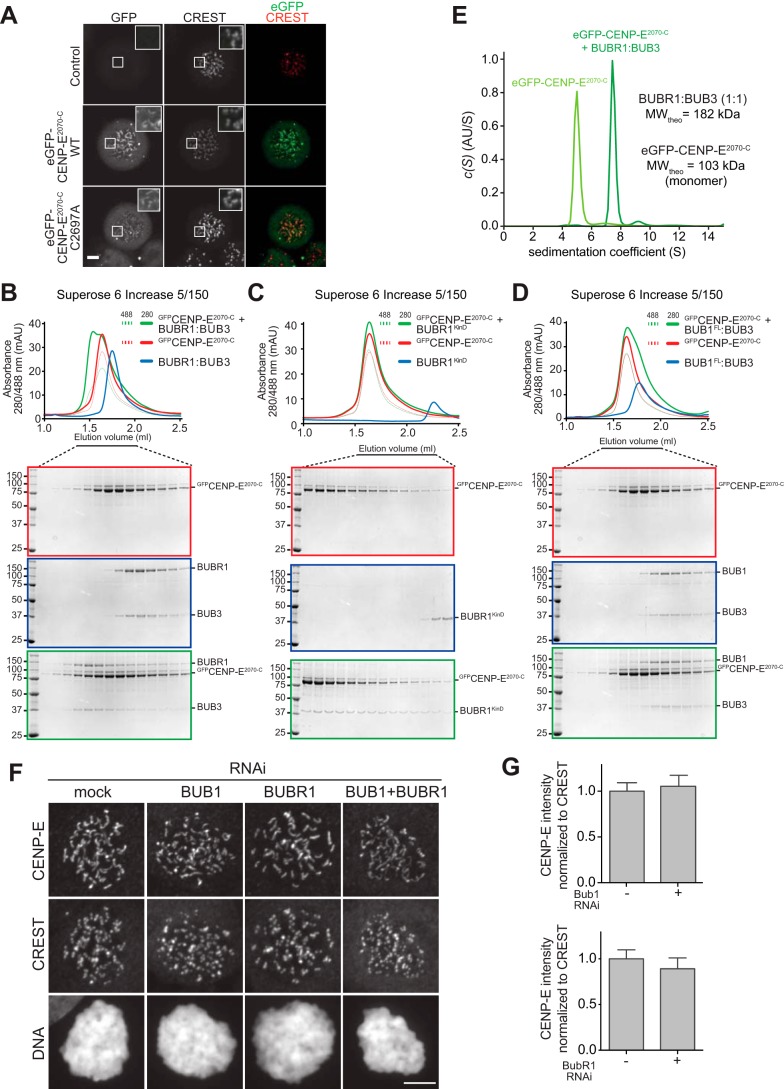
**CENP-E interacts with the BUBR1 pseudo-kinase domain, but BUBR1 is not required for its kinetochore localization.** *A,* representative images of mitotic HeLa cells electroporated with eGFP, eGFP-CENP-E^2070-C^ WT, or eGFP-CENP-E^2070-C^ C2997A mutant (preventing CENP-E farnesylation). *Scale bar,* 5 μm. Both the WT and the unfarnesylated mutant CENP-E constructs localize at kinetochores. *B–D,* elution profiles and SDS-PAGE analysis of SEC experiments of eGFP-CENP-E^2070-C^ with BUBR1/BUB3 complex (*B*), BUBR1 pseudo-kinase domain (KinD) construct (*C*), and BUB1/BUB3 complex (*D*). A shift in elution volume is observed for the BUBR1/BUB3 complex and the BUBR1 pseudo-kinase domain construct, indicative of complex formation. The interaction of CENP-E with BUBR1 is specific, as no shift is observed with the BUB1/BUB3 complex. The elution profiles and SDS-PAGE of eGFP-CENP-E^2070-C^ WT in *B* and *D* and in Fig. 5*E* are the same and are repeated to facilitate the interpretation of the binding experiments with BUB1 and BUBR1 in this figure and CENP-F in Fig. 5*E*. Similarly, and for the same reason, the elution profile and SDS-PAGE of BUBR1/BUB3 in *B* is repeated in Fig. 4*G*, and the elution profile and SDS-PAGE of BUB1/BUB3 in *D* is repeated in Fig. 4*D. E,* sedimentation velocity AUC profiles of eGFP-CENP-E^2070-C^ alone and in complex with BUBR1/BUB3. *AU*, arbitrary units; *MW_theo_*, predicted molecular weight assuming stoichiometry of 1. A reliable estimation of the molecular mass of the proteins in the samples was unsuccessful, likely because of the very elongated and flexible structure of both CENP-E and BUBR1. *F,* representative images of stable Flp-In T-REx cells mock-treated or depleted of endogenous BUB1, BUBR1, or both, showing that CENP-E kinetochore localization is unaffected under any of the conditions. *Scale bar,* 10 μm. *G,* quantification of CENP-E kinetochore levels in cells treated as in *F*. The *graph* shows mean intensity of two independent experiments, and the *error bars* indicate S.E. The mean value for nondepleted cells expressing GFP was set to 1.

Previous studies identified a kinetochore-binding region in residues 2126–2476 of CENP-E (45). By expression in insect cells, we generated a recombinant version of a larger fragment of CENP-E (residues 2070-C) encompassing this region fused to eGFP (eGFP-CENP-E^2070-C^) and purified it to homogeneity. After electroporation in mitotic cells arrested by addition of the microtubule-depolymerizing drug nocodazole, cells were fixed to assess the localization of eGFP-CENP-E^2070-C^. eGFP-CENP-E^2070-C^ localized robustly to mitotic kinetochores (Fig. 3*A*), adopting the typical crescent-like shape previously attributed to the corona (6, 32). An equivalent mutant construct in which Cys-2697 had been mutated to alanine to prevent farnesylation also localized to kinetochores, even if at generally lower levels and without showing a crescent-like distribution, suggesting that farnesylation is not strictly required for kinetochore localization of CENP-E but that it might contribute to an unknown aspect of corona assembly.

In previous Y2H analyses, a CENP-E segment encompassing residues 1958–2662 was found to interact with residues 410–1050 of BUBR1 (37, 45). Even if shorter by more than 100 residues at the N-terminal end, eGFP-CENP-E^2070-C^ interacted directly with the dimeric BUBR1/BUB3 complex in size-exclusion chromatography (SEC) analyses (Fig. 3*B*), as evidenced by the shift in elution volume of both proteins when combined at 16 and 4 μm concentration, respectively. Similar observations were made when we mixed CENP-E^2070-C^ with the BUBR1 pseudokinase domain (KinD, residues 705–1050) (Fig. 3*C*). eGFP-CENP-E^2070-C^, however, did not interact with the paralogous BUB1/BUB3 dimer (Fig. 3*D*). In analytical ultracentrifugation (AUC) sedimentation velocity experiments, in which we monitored the sedimentation of eGFP-CENP-E, addition of unlabeled BUBR1/BUB3 at a 3-fold higher concentration caused a complete shift of eGFP-CENP-E to a species with higher sedimentation coefficient (*s*), indicative of complex formation (Fig. 3*E*). The high frictional ratio of this sample (an indication that the CENP-E structure is very elongated, a consequence of its large coiled-coil content) prevented a quantitative estimate of molecular mass. The analysis, however, strongly suggests that eGFP-CENP-E^2070-C^ adopts the highly elongated conformation of coiled-coils, as shown previously for recombinant full-length CENP-E from *Xenopus laevis* (44). Thus, a minimal segment of CENP-E capable of kinetochore localization interacts directly with the BUBR1/BUB3 complex, and the BUBR1 pseudokinase domain is sufficient for this interaction, at least at the relatively high concentration of the SEC assay. In agreement with our own previous studies (68), BUBR1 did not show any catalytic activity, nor did it become active in presence of eGFP-CENP-E^2070-C^.^4^

In a previous study in egg extracts of *X. laevis*, depletion of BUBR1 was shown to prevent kinetochore localization of CENP-E, an effect that could be rescued by re-addition of WT BUBR1 but not of a deletion mutant lacking the kinase domain (63). CENP-E kinetochore levels were also reduced in DLD-1 cells upon depletion of BUBR1 by RNAi (72).

We therefore asked whether BUBR1 was also important for CENP-E recruitment in HeLa cells. Furthermore, in view of evidence that BUB1 is required for kinetochore recruitment of BUBR1 (70, 71, 73, 86), we also monitored localization of CENP-E upon depletion of BUB1. Contrary to the previous observations in frogs and DLD-1 cells, but in agreement with other studies in HeLa cells (70, 82, 87), RNAi-based depletion of BUB1 or BUBR1 did not result in obvious adverse effects on the kinetochore localization of CENP-E, even after co-depletion of Zwilch (Fig. 3, *F* and *G,* and Fig. S3, *B–E*). These observations suggest that CENP-E, at least in HeLa cells, becomes recruited through a different pathway that does not involve BUB1 and BUBR1. After application of highly specific small-molecule inhibitors, we found CENP-E kinetochore localization to depend on the kinase activity of Aurora B and (to a lower extent) of MPS1, but not of BUB1 or of PLK1 (Fig. S6, *A–D*). The dependence of CENP-E on Aurora B kinase activity for kinetochore localization, together with the central spindle co-localization at anaphase of CENP-E with the chromosome passenger complex (the catalytic subunit of which is Aurora B), leads to speculation that these proteins interact, a hypothesis that will need to be formally tested in the future.

### CENP-F binds to the BUB1 kinase domain

**Figure 4.**
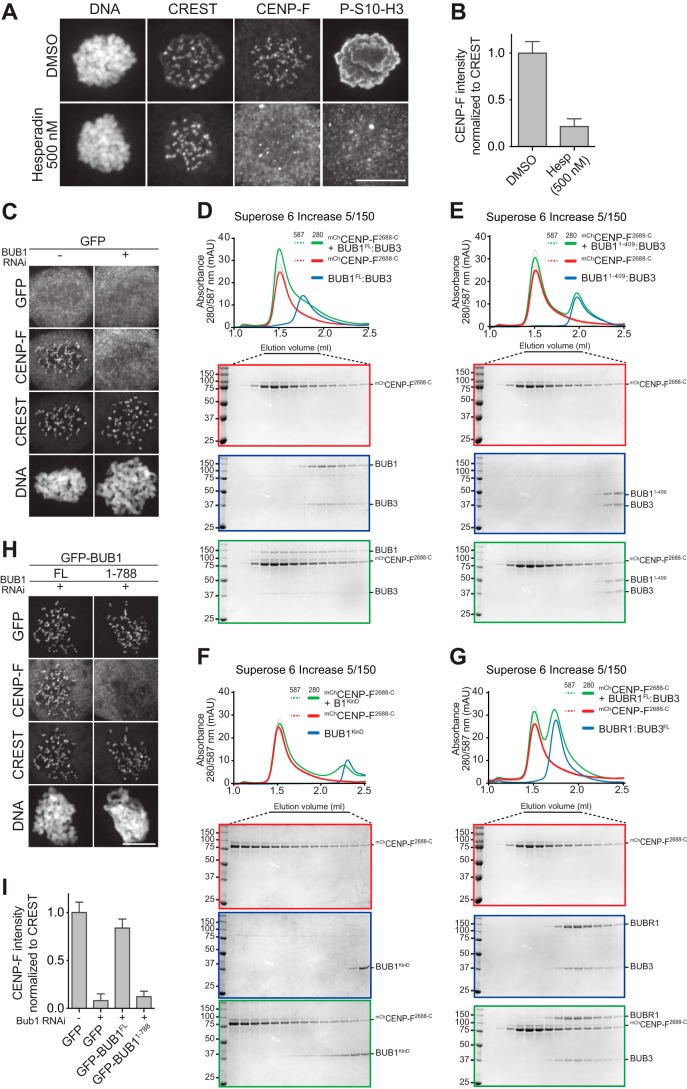
**CENP-F interaction with the BUB1 kinase domain is necessary for its kinetochore localization.** *A,* representative images of mitotic HeLa cells treated with 500 nm hesperadin, showing that CENP-F kinetochore localization is strictly dependent on Aurora B kinase activity. Reduction in P-S10-H3 staining was used as a control for the Aurora B inhibition. *Scale bar,* 10 μm. *B,* quantification of CENP-F kinetochore levels in cells treated as in *A*. The *graph* shows mean intensity of three experiments. The *error bars* indicate S.E., and the mean values for DMSO-treated cells are set to 1. *C,* representative images of GFP-expressing stable HeLa Flp-In T-REx cell lines mock-treated or depleted of BUB1, showing that CENP-F kinetochore recruitment depends on the presence of BUB1 at kinetochores. *D–G,* elution profiles and SDS-PAGE analysis of SEC experiments of mCherry-CENP-F^2688-C^ with the BUB1^FL^/BUB3 complex; *FL*, full-length (*D*), the BUB1(1–409)/BUB3 complex (*E*), the BUB1 kinase domain (KinD) (*F*), and the BUBR1/BUB3 complex (*G*). A shift in the elution volume is only observed for the BUB1 constructs containing the C-terminal kinase domain (*D* and *F*). The interaction of CENP-F with BUB1 is specific, as no shift is observed for the BUBR1/BUB3 complex (*G*). The elution profile and SDS-PAGE of BUBR1/BUB3 in *G* is the same shown in Fig. 3*B*. The elution profile and SDS-PAGE of BUB1/BUB3 in *D* is the same shown in Fig. 3*D*. The elution profiles and SDS-PAGE of mCherry-CENP-F^2688-C^ in *D, E,* and *G* and in Fig. 5*E* are the same and were included to facilitate the interpretation of binding experiments by inclusion of elution references. *H,* representative images of stable HeLa Flp-In T-REx cell lines depleted of endogenous BUB1 and expressing GFP-BUB1 full-length or lacking the kinase domain (BUB1(1–788)). CENP-F kinetochore recruitment depends on the BUB1 kinase domain, as BUB1(1–788) does not rescue CENP-F localization, whereas full-length BUB1 does. *Scale bar,* 10 μm. *I,* quantification of CENP-F kinetochore levels in cells of *C* and *H*. The *graph* shows mean intensity of three independent experiments; the *error bars* indicate S.E. The mean value for nondepleted cells expressing GFP is set to 1.

Next, we asked how CENP-F becomes recruited to kinetochores. CENP-F recruitment was strictly dependent on the kinase activity of Aurora B, partly dependent on that of MPS1 and PLK1, and not dependent on that of BUB1 (Fig. 4*A* and Fig. S7). This pattern of kinetochore localization is reminiscent of that of BUB1, which has been previously shown to be important for CENP-F kinetochore recruitment (69–72). In agreement with these previous studies, RNAi-based depletion of BUB1 resulted in complete ablation of CENP-F from kinetochores (Fig. 4*C*, see *I* for quantification).

By expression in insect cells, we generated a recombinant fragment of CENP-F encompassing its previously identified kinetochore-binding domain (residues 2688-C) (51, 53, 55) fused to an N-terminal mCherry tag. In SEC experiments, mCherry-CENP-F^2688-C^ bound BUB1/BUB3 directly, as indicated by its altered elution volume in the presence of the CENP-F construct (Fig. 4*D*; note that mCherry-CENP-F^2688-C^ is highly elongated, as shown below, and therefore its hydrodynamic radius, which determines elution volume in SEC experiments, is unlikely to change as a result of an interaction with BUB1/BUB3). In contrast, mCherry-CENP-F^2688-C^ failed to interact with BUB1(1–409)/BUB3, where the BUB1 deletion mutant BUB1(1–409) lacks a central region of BUB1 and its kinase domain (Fig. 4*E*). Indeed, mCherry-CENP-F^2688-C^ bound the BUB1 kinase domain (BUB1^KinD^, residues 724–1085; Fig. 4*F*). Conversely, mCherry-CENP-F^2688-C^ did not interact with the BUBR1/BUB3 complex (Fig. 4*G*). Thus, the kinetochore-targeting domain of CENP-F interacts directly with the BUB1/BUB3 complex, and the kinase domain appears to be necessary and partly sufficient for this interaction.

In agreement with these *in vitro* findings, we observed robust kinetochore localization of endogenous CENP-F in HeLa cells previously depleted of BUB1 by RNAi and expressing an RNAi-resistant GFP-BUB1 transgene, whereas CENP-F kinetochore localization appeared entirely compromised in BUB1-depleted cells expressing GFP-BUB1(1–788), which lacks exclusively the BUB1 kinase domain (Fig. 4, *H* and *I*). Collectively, our observations indicate that the kinase domain of BUB1 is sufficient for a direct interaction with CENP-F *in vitro* and is necessary for kinetochore recruitment of CENP-F in HeLa cells. A very recent study identified a similar requirement for the kinase domain of BUB1 in kinetochore recruitment of CENP-F in HAP1 cells (69).

### CENP-F dimerization is important for BUB1 binding

**Figure 5.**
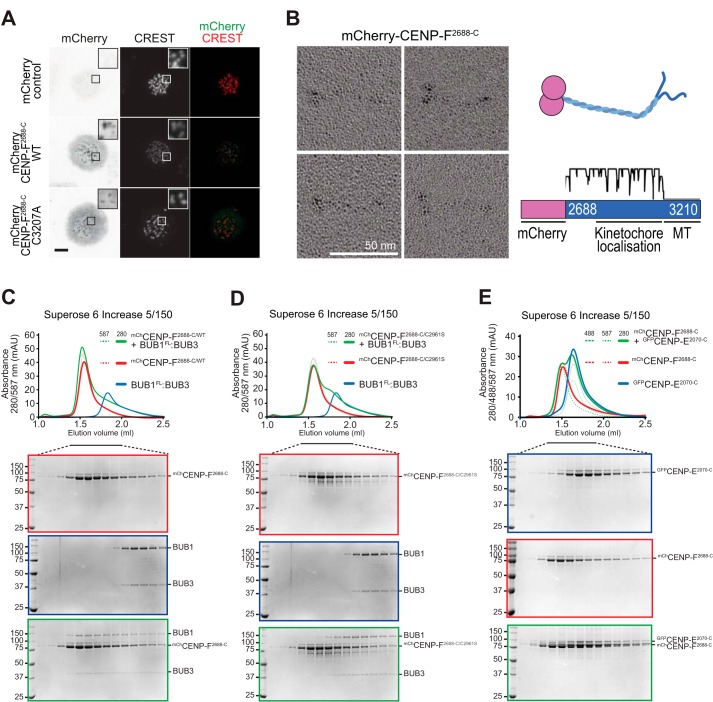
**Requirements for CENP-F kinetochore localization.** *A,* representative images of mitotic HeLa cells electroporated with mCherry, mCherry-CENP-F^2688-C^ WT, or mCherry-CENP-F^2688-C/C3207A^ (farnesylation mutant). *Scale bar,* 5 μm. As for CENP-E, both the WT and the unfarnesylated mutant CENP-F constructs localize at kinetochore. *B,* mCherry-CENP-F^2688-C^ sample was visualized by EM after glycerol spraying and low-angle platinum shadowing (*right panel*). The elongated shape of the observed particles is consistent with the secondary structure expected for the mCherry-tag coiled-coil construct (*right panel*). *C* and *D,* SEC elution profiles and SDS-PAGE analysis of binding experiments with 16 μm each of mCherry-CENP-F^2688-C^ WT (*C*) or C2961S mutant (*D*) and 4 μm BUB1^FL^/BUB3 complex. The shift in elution volume of BUB1/BUB3 is observed with both WT and mutant CENP-F, but it is significantly less pronounced for the CENP-F mutant, suggesting that the C2961S mutation reduces the affinity of CENP-F for the BUB1 kinase domain without completely abolishing it. *E,* SEC elution profile and SDS-PAGE analysis of a binding experiment with 16 μm each of mCherry-CENP-F^2688-C^ and eGFP-CENP-E^2070-C^. No shift is observed, indicating that the tested constructs do not interact. The elution profile and SDS-PAGE of eGFP-CENP-E^2070-C^ WT in *E* is the same already shown in Fig. 3, *B* and *D*. Similarly, the elution profiles and SDS-PAGE of mCherry-CENP-F^2688-C^ in *E* is the same as in Fig. 4, *D, E,* and *G*. Similarly, the elution profiles and SDS-PAGE of BUB1/BUB3 in *C* and *D* are the same. These repetitions were included to facilitate the interpretation of binding experiments by inclusion of elution references.

The mCherry-CENP-F^2688-C^ construct that interacted with BUB1/BUB3 in SEC experiments also localized to mitotic kinetochores when electroporated in HeLa cells (Fig. 5*A*). A farnesylation mutant of this construct on which Cys-3207 had been mutated to alanine retained kinetochore localization, although not as robustly as the WT counterpart (Fig. 5*A*). This result suggests that farnesylation is not strictly required for kinetochore recruitment of CENP-F, as already observed with CENP-E (Fig. 3*A*). Collectively, our results with electroporated farnesylation mutants of CENP-E and CENP-F are in agreement with results obtained with farnesyltransferase inhibitors, in which only partial repression of kinetochore recruitment of CENP-E and CENP-F was observed (61).

By rotary shadowing electron microscopy (EM), which is particularly suited to the study of elongated coiled-coil proteins, mCherry-CENP-F^2688-C^ had the appearance of a highly elongated (∼40 nm) rod. Most likely, the latter corresponds to a predicted coiled-coil positioned between residues 2688 and ∼3000, flanked on one side by two globular domains, most likely corresponding to mCherry, and on the other side by disordered fragments corresponding to the last ∼200 residues and containing the C-terminal microtubule-binding domains (Fig. 5*B*) (56–58). Collectively, these observations suggest that CENP-F^2688-C^ contains a parallel dimeric coiled-coil, like the one previously identified in CENP-E (44).

On the basis of previous studies implicating Cys-2864 in kinetochore recruitment of mouse CENP-F (53), we generated a mutant version of human mCherry-CENP-F^2688-C^ in which the equivalent residue, Cys-2961, was mutated to serine (our residue numbering is in accordance with the 3210-residue human CENP-F sequence in Uniprot). Although WT mCherry-CENP-F^2688-C^ interacted with BUB1/BUB3 in SEC experiments, as already shown, the interaction was at least partially impaired when the mCherry-CENP-F^2688-C/C2961S^ mutant was analyzed, confirming the role of Cys-2961 in kinetochore localization and implicating this residue in the interaction with BUB1 (Fig. 5, *C* and *D*). Furthermore, mCherry-CENP-F^2688-C^ did not interact with GFP-CENP-E^2070-C^ in SEC experiments, as predicted based on previous work identifying the CENP-E-binding region of CENP-F within a segment (residues 1804–2104) that precedes and is not included in CENP-F^2688-C^ (Fig. 5*E*) (37, 45).

**Figure 6.**
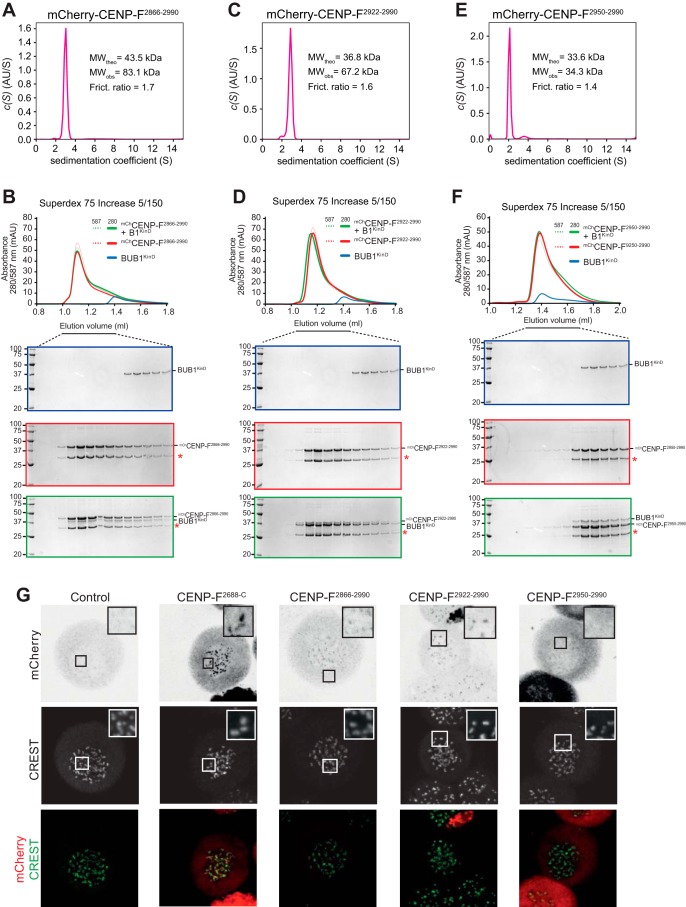
**Identification of a minimal CENP-F construct for binding to BUB1.** *A,* sedimentation velocity AUC results of the indicated mCherry-CENP-F constructs. *MW_obs_*, observed molecular weight; *MW_theo_*, the predicted molecular weight of the monomer; *Frict. ratio* denotes the frictional ratio; *AU*, arbitrary units. mCherry-CENP-F(2866–2990) forms a dimer. *B,* elution profiles and SDS-PAGE analysis of SEC experiments of the BUB1 kinase domain (*KinD*) with mCherry-CENP-F(2866–2990). The shift in elution volume of BUB1^KinD^ indicated binding. The *red asterisk* indicated a breakdown product of mCherry that is produced during boiling in sample buffer. *C,* as in *A* but with the CENP-F(2922–2990) construct, which is also dimeric. *D,* as in *B* but with the CENP-F(2922–2990) construct. Also in this case, an interaction with the kinase domain of CENP-F is clearly discernible. *E,* as in *A* but with the CENP-F(2950–2990) construct, which is monomeric. *F,* as in *B* but with the CENP-F(2950–2990) construct. In this case, no interaction with the kinase domain of CENP-F is discernible. The elution profile and SDS-PAGE of the BUB1 kinase domain in *B, D,* and *F* are the same and were included to facilitate the interpretation of binding experiments by inclusion of elution references. *G,* representative images of mitotic HeLa cells electroporated with the indicated constructs. mCherry-CENP-F^2688-C^ (positive control), mCherry-CENP-F(2866–2990) and mCherry-CENP-F(2922–2990) localized to kinetochores, whereas mCherry (negative control) and mCherry-CENP-F(2950–2990) did not. *Scale bar,* 5 μm.

The effects of the Cys-2961 mutation made us ask whether we could identify a minimal BUB1-binding domain of CENP-F. For this, we further trimmed CENP-F. CENP-F(2866–2990), which is entirely encompassed within the predicted coiled-coil of CENP-F, appeared dimeric by sedimentation velocity AUC and retained the ability to bind to the BUB1 kinase domain in a SEC experiment (Fig. 6, *A* and *B*). Similar results were obtained with an even shorter CENP-F fragment, CENP-F(2922–2990) (Fig. 6, *C* and *D*). CENP-F(2950–2990), in contrast, appeared monomeric in AUC runs and was unable to interact with the BUB1 kinase domain (Fig. 6, *E* and *F*). These observations do not allow us to resolve whether impaired binding to the BUB1 kinase domain CENP-F(2950–2990) is due to loss of dimerization or to trimming of residues directly involved in the interaction, but we identify CENP-F(2922–2990) as a minimal BUB1-binding fragment of CENP-F. In agreement with these observations, mCherry-CENP-F(2866–2990) and mCherry-CENP-F(2922–2990) labeled kinetochores after electroporation in HeLa cells, albeit weakly compared with mCherry-CENP-F^2866-C^, whereas mCherry-CENP-F(2950–2990) did not localize to kinetochores (Fig. 6*G*).

### BUBR1/BUB3, BUB1/BUB3, CENP-E, and CENP-F in a single complex

**Figure 7.**
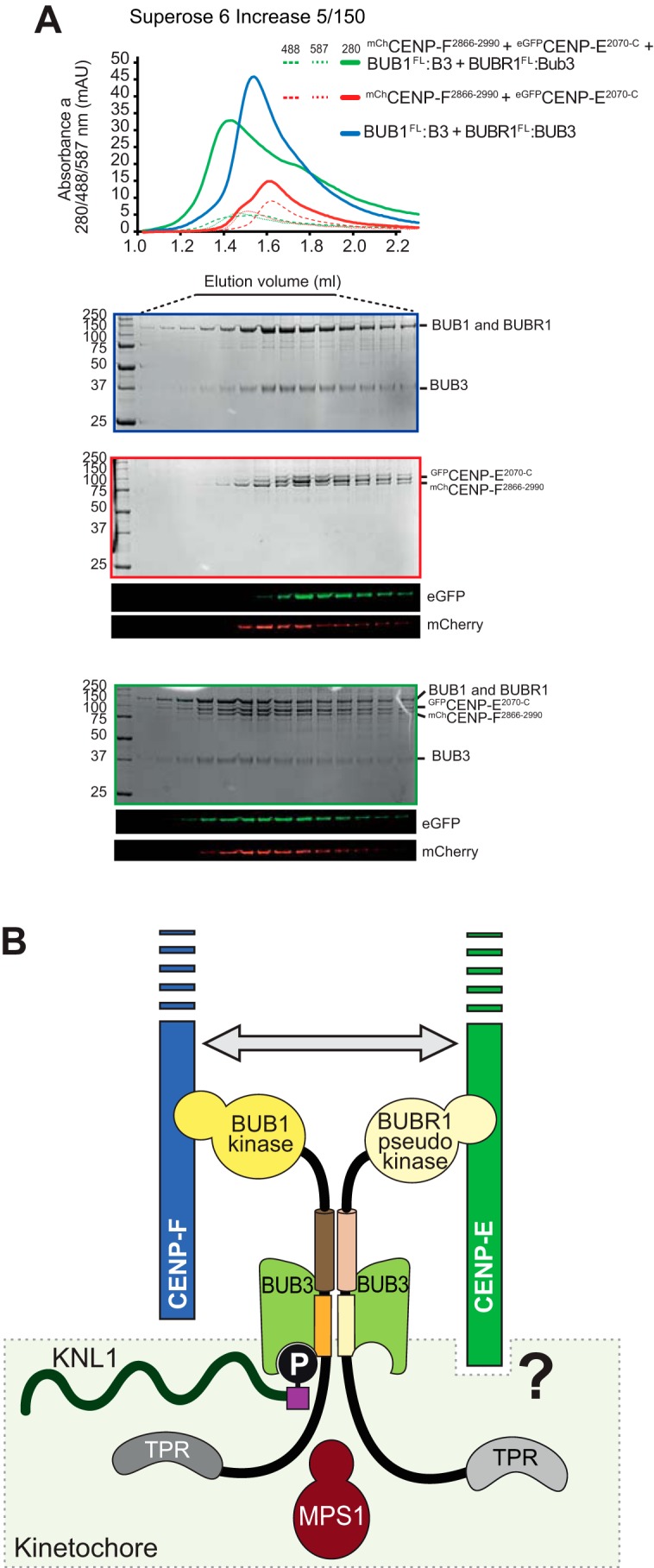
**Schematic of the interactions of BUB1 and BUBR1 paralogs.** *A,* elution profiles and SDS-PAGE analysis of SEC experiments with BUB1/BUB3, BUBR1/BUB3, eGFP-CENP-E^2070-C^, and mCherry-CENP-F^2688-C^, each at 6 μm loading concentration. The elution of eGFP-CENP-E^2070-C^ and mCherry-CENP-F^2688-C^ was additionally monitored through their fluorescence. *B,* schematic summarizes the interactions occurring at kinetochores between CENP-E, CENP-F, BUB1, and BUBR1. BUB1 is recruited to the kinetochore subunit Knl1 (see Introduction) after phosphorylation by the SAC kinase Mps1. There, BUB1 recruits BUBR1 through a pseudo-dimeric interaction (64). CENP-F kinetochore localization strictly depends on BUB1, whereas CENP-E recruitment requires a wider and still uncharacterized network of interactions, indicated by a question mark. RZZ and MAD1 recruitment, as well as the corona expansion, appear to be independent from CENP-E and CENP-F, and are not shown. An interaction of CENP-E and CENP-F has also been identified (*gray arrow*), but is not sufficient for CENP-F localization in absence of BUB1.

Previously, we have reported that the BUBR1/BUB3 and BUB1/BUB3 complexes interact directly and that this interaction is responsible for kinetochore recruitment of BUBR1/BUB3 to phosphorylated MELT (Met-Glu-Leu-Thr) motifs on the kinetochore receptor KNL1 (68, 73, 74). The interaction of BUBR1/BUB3 and BUB1/BUB3 probably reflects the ancient homodimerization of a singleton that preceded the duplication of the BUBR1 and BUB1 paralogs, because it involves equivalent structural domains in BUB1 and BUBR1, comprising the BUB3-binding domain and a subsequent predicted helical domain (73, 74). We asked whether the interaction of BUBR1/BUB3 and BUB1/BUB3 was compatible with their respective interactions with CENP-E and CENP-F, respectively. As already shown in Fig. 5*E*, mCherry-CENP-F(2866–2990) and eGFP-CENP-E^2070-C^ eluted independently in SEC experiments, showing that they do not interact (Fig. 7*A*). However, mCherry-CENP-F^2688-C^ and eGFP-CENP-E^2070-C^ co-eluted when BUBR1/BUB3 and BUB1/BUB3 were also added, indicating that all individual interactions are preserved when all binding species are combined (Fig. 7*A*).

## Conclusions

Previous studies on human BUB1 and BUBR1, including our own work, demonstrated that these paralogs sub-functionalized in various ways as follows: 1) the selective inactivation of the kinase domain in BUBR1; 2) the development of phospho-amino acid recognition modules that contribute to the ability of BUB3 to recognize distinct substrates; and 3) the interaction with distinct binding partners that subtend to distinct functions in chromosome alignment and mitotic checkpoint signaling (summarized in Fig. 7*B*) (67, 73–75, 88).

In this study, we report an additional aspect of this sub-functionalization and show that the kinase domains of BUBR1 and BUB1 interact, respectively, with C-terminal regions of CENP-E and CENP-F that encompass the kinetochore-targeting domains of these proteins. Both interactions are direct and were reproduced with recombinant proteins. Neither interaction appears to be crucially required for downstream signaling events. In the BUBR1 case, it appears well established that deletion of the pseudokinase domain in human cells is compatible with its functions in the SAC as a subunit of the MCC, the SAC effector (64). We and others have also shown that depletion of BUBR1 does not affect the CENP-E kinetochore localization in HeLa cells (this study and Refs. 82, 87). In *Xenopus* egg extracts, however, the kinase domain of BUBR1 has been implicated in CENP-E recruitment, and this interaction has been shown to be important for SAC silencing (63). Given the very complex evolutionary history of the BUB1 and BUBR1 paralogs or of the singleton from which they originate, these apparent differences may genuinely reflect different evolutionary paths in these organisms (67, 88). A question for future work that this study raises regards the detailed mechanism of kinetochore recruitment of CENP-E in human cells, which remains unknown.

The role of BUB1 in CENP-F kinetochore recruitment was already established in previous work (69, 70, 72, 89), and a very recent study implicated the BUB1 kinase domain in CENP-F kinetochore recruitment (69). Here, we have extended this previous study by showing that the interaction of the BUB1 kinase domain and CENP-F is direct and by identifying a minimal CENP-F domain involved in this interaction and capable of kinetochore localization. Our identification of a minimal kinetochore-targeting domain of HsCENP-F within residues 2922–2990 agrees with a previous study that made use of CENP-F deletion mutants (53). It also agrees with another study (89) that identified distinct binding domains in CENP-F for nuclear envelope and for kinetochore localization. Specifically, residues 2655-2860 of mouse CENP-F (corresponding to residues 2866–3072 of HsCENP-F) were sufficient for kinetochore recruitment (89). Within this fragment, a specialized N-terminal sub-domain (residues 2655–2723, corresponding to HsCENP-F residues 2866–2933) bound Nup133, a subunit of the nuclear pore complex Nup107/Nup160, and mediated CENP-F recruitment to the nuclear envelope shortly before mitosis, but it was not required for kinetochore recruitment. A specialized C-terminal sub-domain (residues 2724–2860, corresponding to HsCENP-F residues 2934–3072), in contrast, was required for kinetochore recruitment (89). As previous studies identified CENP-E as a binding partner of CENP-F (37, 45, 63), CENP-E may reinforce binding of CENP-F to kinetochores, as shown by Berto *et al.* (89). However, there is now sufficient evidence to conclude that this interaction is clearly not sufficient to promote stable binding of CENP-F to kinetochores in absence of BUB1.

Our observation that CENP-F depletion results in very mild chromosome alignment defects, in line with other reports (69), is surprising. CENP-F has been implicated in dynein recruitment and regulation through a pathway involving Nde1, Ndel1, and Lis1, the product of the lissencephaly type 1 gene (77–79, 90, 91). However, CENP-F is not sufficient for stable kinetochore recruitment of dynein, as it does not seem to be able to complement the very strong reduction or loss of kinetochore dynein in cells depleted of the RZZ complex or Spindly (19, 21, 24). The latter therefore appears to be the dominant factor in dynein recruitment to kinetochores. It is plausible, however, that the consequences of CENP-F depletion are exacerbated by concomitant depletion of RZZ (91).

As already discussed in the Introduction, various common features of CENP-E and CENP-F support the speculation that they are distantly related paralogs. Their ability to interact with the kinase domains of BUBR1 and BUB1, themselves paralogs, lends strong further credit to this hypothesis. Apparent lack of strong functional consequences from disrupting these interactions may indicate that they may have become vestigial in some species. It is also possible, however, that these interactions play more important roles during development or in specific cell types. The dissection described here will allow testing this hypothesis in future work.

## Experimental procedures

### Plasmids

The codon-optimized cDNAs of *Homo sapiens* CENP-E (Q02224) and *CENP-F* (P49454) were synthesized at GeneWiz. *CENP-E* and *CENP-F* constructs were subcloned, respectively, in pLIB-eGFP and pLIB-mCherry or pET-mCherry, modified versions of the pLIB (92) and pET-28 vectors for expression of proteins with N-terminal PreScission-cleavable His_6_-eGFP or His_6_-mCherry tags. Site-directed mutagenesis was performed by PCR (93). All constructs were sequence-verified. The vectors for the co-expression of full-length BUB1 and BUBR1 proteins with BUB3, as well as that for the BUB1 and BUBR1 constructs were described previously (68, 73).

### Protein expression and purification

Expression and purification of eGFP-CENP-E^2070-C^ and mCherry-CENP-F^2866-C^ WT and mutants were carried out in insect cells using a pBig system (92). Baculoviruses were generated in Sf9 cells and used to infect Tnao38 cells for 48–96 h at 27 °C. Cells were collected by centrifugation, washed in PBS, and then frozen at −80 °C. CENP-E–expressing cell pellets were resuspended in lysis buffer (50 mm sodium phosphate buffer, pH 8.0, 500 mm NaCl, 5% (w/v) glycerol, and 0.5 mm TCEP) supplemented with protease inhibitor mixture, lysed by sonication, and cleared by centrifugation at 100,000 × *g* at 4 °C. The supernatant was filtered and loaded on a 5-ml HisTrap FF column (GE Healthcare) equilibrated in lysis buffer. After washing with lysis buffer, the protein was eluted with a linear gradient of 0–250 mm imidazole in 10 column volumes. The fractions of interest were pooled, concentrated with a 50-kDa cutoff Amicon concentrator (Millipore), and loaded onto a Superose 6 Increase 10/300 (GE Healthcare) equilibrated in SEC buffer (50 mm Hepes, pH 8.0, 200 mm NaCl, 5% (w/v) glycerol, and 0.5 mm TCEP). CENP-E–containing fractions were concentrated, flash-frozen in liquid nitrogen, and stored at −80 °C. The purification protocol for the mCherry-CENP-F^2866-C^ constructs is identical to that of eGFP-CENP-E^2070-C^, but the lysis and the SEC buffers were at pH 7.0.

The constructs mCherry-CENP-F(2866–2990), mCherry-CENP-F(2922–2990), and mCherry-CENP-F(2950–2990) were expressed in *Escherichia coli* BL21 (DE3) RP plus cells grown at 37 °C to *A*_600_ = 2 and then induced with 0.25 mm IPTG for 16 h at 25 °C. Cell were collected by centrifugation, washed in PBS, and then frozen at −80 °C. Cell pellets were resuspended in lysis buffer (50 mm sodium phosphate buffer, pH 7.5, 500 mm NaCl, 5% (w/v) glycerol, and 2 mm β-mercaptoethanol) supplemented with protease inhibitor mixture, lysed by sonication, and cleared by centrifugation at 70,000 × *g* at 4 °C. The supernatant was filtered and loaded on a 5-ml HisTrap FF column (GE Healthcare) equilibrated in lysis buffer. After washing with lysis buffer, the protein was eluted with a linear gradient of 0–500 mm imidazole in 10 column volumes. The fractions of interest were pooled, concentrated with a 10-kDa cutoff Amicon concentrator (Millipore), and loaded onto a HiLoad Superdex 75 16/60 (GE Healthcare) equilibrated in SEC buffer (50 mm sodium phosphate buffer, pH 7.0, 200 mm NaCl, 5% (w/v) glycerol, and 1 mm TCEP). Expression and purification of BUB1 and BUBR1 constructs, as well as of BUB1/BUB3 and BUBR1/BUB3 complexes, were carried out as described previously (68, 73).

### Analytical SEC analysis

4 μm BUB1 and BUBR1 protein constructs or BUB1/BUB3 and BUBR1/BUB3 complexes were mixed with 16 μm CENP-E and CENP-F proteins, respectively, in a 30-μl final volume. Analytical size-exclusion chromatography was carried out at 4 °C on a Superose 6 5/150 or Superdex 75 5/150 in a buffer containing 50 mm Hepes, pH 8.0, 100 mm NaCl, 5% (w/v) glycerol, and 0.5 mm TCEP at a flow rate of 0.12 ml/min on an ÄKTA Microsystem. Elution of proteins was monitored at 280, 488 (eGFP-tag), and 587 nm (mCherry-tag). 50-μl fractions were collected and analyzed by SDS-PAGE and Coomassie Blue staining.

AUC was performed at 42,000 rpm at 20 °C in a Beckman XL-A ultracentrifuge. Protein samples were loaded into standard double-sector centerpieces. The cells were scanned every minute, and 500 scans were recorded for every sample. 6 μm mCherry-CENP-F(2866–2990), mCherry-CENP-F(2922–2990), and mCherry-CENP-F(2950–2990) were scanned at 587 nm. 7 μm eGFP-CENP-E^2070-C^ alone or mixed with 21 μm BUBR1/BUB3 were instead scanned at 488 nm. Data were analyzed using the program SEDFIT (94) with the model of continuous *c*(*s*) distribution. The partial specific volumes of the proteins, buffer density, and buffer viscosity were estimated using the program SEDNTERP. Data figures were generated using the program GUSSI (95).

### Protein electroporation

For eGFP-CENP-E protein electroporation, HeLa cells were arrested in G_2_ with a 9 μm RO-3306 treatment for 15 h (Millipore) and then released into mitosis for 3 h in presence of 3.3 μm nocodazole. Mitotic cells were then collected by mitotic shake-off, washed with PBS, and counted. Approximately 3 × 10^6^ cells were then electroporated (Neon transfection system kit, ThermoFisher Scientific) with 10 μm eGFP-CENP-E. Following electroporation, cells were allowed to recover in media with 3.3 μm nocodazole for 4 h and then fixed and prepared for immunofluorescence analysis. For mCherry-CENP-F protein electroporation, HeLa cells were treated for 16 h with 0.33 μm nocodazole (Sigma) to synchronize cells in mitosis. Mitotic cells were then collected by mitotic shake-off, washed with PBS, and counted. Approximately 3 × 10^6^ cells were then electroporated with 5 μm mCherry-CENP-F. Following electroporation, cells were allowed to recover in media with 3.3 μm nocodazole for 4 h and then fixed and prepared for immunofluorescence analysis.

### Low-angle metal shadowing and EM

mCherry-CENP-F^2688-C^ fractions from the elution peak of an analytical size-exclusion chromatography column were diluted 1:1 with spraying buffer (200 mm ammonium acetate and 60% glycerol) and air-sprayed as described (96, 97) onto freshly cleaved mica pieces of ∼2 × 3 mm (V1 quality, Plano GmbH). Specimens were mounted and dried in a MED020 high-vacuum metal coater (Bal-Tec^TM^). A platinum layer of ∼1 nm and a 7-nm carbon support layer were evaporated subsequently onto the rotating specimen at angles of 6–7 and 45°, respectively. Platinum/carbon replicas were released from the mica on water, captured by freshly glow-discharged 400-mesh palladium/copper grids (Plano GmbH), and visualized using a LaB6 equipped JEM-1400 transmission electron microscope (JEOL) operated at 120 kV. Images were recorded at a nominal magnification of ×60,000 on a 4000 × 4000 CCD camera F416 (TVIPS), resulting in 0.18 nm per pixel.

### Mammalian plasmids

Plasmids were derived from the *pCDNA5/FRT/TO–eGFP–IRES*, a previously modified version (98) of the *pCDNA5/FRT/TO* vector (Invitrogen). To create N-terminally-tagged eGFP-BUB1 truncation constructs, the *BUB1* sequence was obtained by PCR amplification from the previously generated *pCDNA5/FRT/TO–eGFP–BUB1–IRES* vector (98) and subcloned in-frame with the GFP-tag. All *BUB1* constructs were RNAi-resistant (99). *pCDNA5/FRT/TO*-based plasmids were used for generation of stable cell lines. All plasmids were verified by sequencing.

### Cell culture and transfection

HeLa cells were grown in DMEM (PAN Biotech) supplemented with 10% FBS (Clontech), penicillin and streptomycin (GIBCO), and 2 mm l-glutamine (PAN Biotech). Flp-In T-REx HeLa cells used to generate stable doxycycline-inducible cell lines were a gift from S. S. Taylor (University of Manchester, Manchester, UK). Flp-In T-REx host cell lines were maintained in DMEM with 10% tetracycline-free FBS (Clontech) supplemented with 50 μg/ml Zeocin (Invitrogen). Flp-In T-REx HeLa expression cell lines were generated as described previously (98). Briefly, Flp-In T-Rex HeLa host cells were co-transfected at a ratio of 9:1 (w/w) *pOG44:pcDNA5/FRT/TO* expression plasmid using X-tremeGene transfection agent (Roche Applied Science). 48 h after transfection, Flp-In T-Rex HeLa expression cell lines were put under selection for 2 weeks in DMEM with 10% tetracycline-free FBS (Invitrogen) supplemented with 250 μg/ml hygromycin (Roche Applied Science) and 5 μg/ml blasticidin (ICN Chemicals). The resulting foci were pooled and tested for expression. Gene expression was induced by addition of 0.5 μg/ml doxycycline (Sigma) for 24 h.

siBUB1 (Dharmacon, 5′-GGUUGCCAACACAAGUUCU-3′) or siBUBR1 (Dharmacon, 5′-CGGGCAUUUGAAUAUGAAA-3′) duplexes were transfected with Lipofectamine 2000 (Invitrogen) at 50 nm for 24 h. siCENP-E (Dharmacon, 5′-AAGGCUACAAUGGUACUAUAU-3′) and siCENP-F (Dharmacon, 5′-CAAAGACCGGUGUUACCAAG-3′ and 5′-AAGAGAAGACCCCAAGUCAUC-3′) duplexes were transfected at 60 nm with LipofectamineRNAiMAX (Invitrogen) for 24 h. siZwilch (SMART pool from Dharmacon, L-019377-00-0005) duplexes were transfected with LipofectamineRNAiMAX at 120 nm for 72 h.

### Immunoblotting

To generate mitotic populations for immunoblotting experiments, cells were treated with 330 nm nocodazole for 16 h. Mitotic cells were then harvested by shake off and lysed in lysis buffer (150 mm KCl, 75 mm Hepes, pH 7.5, 1.5 mm EGTA, 1.5 mm MgCl_2_, 10% glycerol, and 0.5% Triton X-100 supplemented with protease inhibitor mixture (Serva) and PhosSTOP phosphatase inhibitors (Roche Applied Science)). Cleared cell lysates were resuspended in sample buffer, boiled, and analyzed by SDS-PAGE using 3–8% gradient gels (NuPAGE® Tris acetate gels, Life Technologies, Inc.) and Western blotting. The following antibodies were used: anti-CENP-E (rabbit, ab133583, 1:500), anti-CENP-F (rabbit, Novus NB500-101, 1:500) and anti-tubulin (mouse, Sigma T9026, 1:10,000). Secondary antibodies were anti-mouse (Amersham Biosciences) or anti-rabbit (Amersham Biosciences) affinity-purified with horseradish peroxidase conjugate (working dilution 1:10,000). After incubation with ECL Western blotting system (GE Healthcare), images were acquired with the ChemiDoc^TM^ MP Imaging System (Bio-Rad) in 16-bit TIFF format. Images were cropped and converted to 8-bit using Image J software (National Institutes of Health). Brightness and contrast were adjusted using Photoshop CS5 (Adobe).

### Live cell imaging

Cells were plated on a 24-well μ-Plate (Ibidi®). The medium was changed to CO_2_ Independent Medium (Gibco®) 6 h before filming. DNA was stained by addition of the SiR-Hoechst-647 Dye (Spirochrome) to the medium 1 h before imaging. Cells were imaged every 5–10 min in a heated chamber (37 °C) on a 3i Marianas^TM^ system (Intelligent Imaging Innovations, Inc.) equipped with Axio Observer Z1 microscope (Zeiss), Plan-Apochromat ×40/1.4NA oil objective, M27 with DIC III Prism (Zeiss), Orca Flash 4.0 sCMOS Camera (Hamamatsu), and controlled by Slidebook Software 6.0 (Intelligent Imaging Innovations, Inc).

### Immunofluorescence

HeLa cells and Flp-In T-REx HeLa cells were grown on coverslips precoated with poly-d-lysine (Millipore, 15 μg/ml) and poly-l-lysine (Sigma), respectively. Asynchronously growing cells or cells that were arrested in prometaphase by the addition of nocodazole (Sigma) were fixed using 4% paraformaldehyde. Cells were stained as follows: BUB1 (mouse, ab54893, 1:400); BUBR1 (rabbit, Bethyl A300-386A-1, 1:1000); tubulin (mouse, DM1a Sigma, 1:500); CENP-E (mouse, ab5093, 1:200); CENP-F (rabbit, Novus NB500-101, 1:300); Zwilch (rabbit, made in-house, SI520, 1:900); MAD1 labeled with AlexaFluor-488 (mouse, made in-house, Clone BB3-8, 1:200); pT232-AurB (rabbit, Rockland 660-401-667, 1:2000); Plk1 (mouse, ab17057, 1:300); pS10H3 (mouse, ab14955, 1:3000); pT121 H2A (rabbit, active motif 39391, 1:2000); and CREST/anti-centromere antibodies (Antibodies, Inc., 1:100), diluted in 2% BSA/PBS for 1.5 h.

For testing the effect of various kinase inhibitors on CENP-E and CENP-F kinetochore localization, the protocol was adapted in the following way. Cells were pre-permeabilized with 0.5% Triton X-100 solution in PHEM (Pipes, Hepes, EGTA, MgCl_2_) buffer for 2 min before fixation with 4% paraformaldehyde/PHEM for 15 min. After blocking the cells with 3% BSA/PHEM buffer supplemented with 0.1% Triton X-100, they were incubated at room temperature for 1–2 h with primary antibodies diluted in blocking buffer. Washing steps were performed in PHEM-T buffer.

Goat anti-human (Invitrogen), goat anti-mouse (Jackson ImmunoResearch), and goat anti-rabbit (Jackson ImmunoResearch) fluorescently labeled antibodies were used as secondary antibodies. DNA was stained with 0.5 μg/ml DAPI (Serva), and coverslips were mounted with Mowiol mounting media (Calbiochem). Cells were imaged at room temperature using a spinning disk confocal device on the 3i Marianas^TM^ system equipped with an Axio Observer Z1 microscope (Zeiss), a CSU-X1 confocal scanner unit (Yokogawa Electric Corp.), Plan-Apochromat ×63 or ×100/1.4NA Oil Objectives (Zeiss) and Orca Flash 4.0 sCMOS Camera (Hamamatsu). Images were acquired as z-sections at 0.27 μm. Images were converted into maximal intensity projections, exported, and converted into 8-bit tiff files. Quantification of kinetochore signals was performed on unmodified 16-bit z-series images using Imaris 7.3.4 32-bit software (Bitplane). After background subtraction, all signals were normalized to CREST. At least 117 kinetochores were analyzed per condition. Measurements were exported in Excel (Microsoft) and graphed with GraphPad Prism 6.0 (GraphPad Software).

### Cell synchronization

To test the effect of various kinase activities on CENP-E and CENP-F kinetochore localization, cells were synchronized using a double thymidine arrest. Cells were released from the first 18-h thymidine (2 mm; Sigma) block by washing them with fresh pre-warmed media several times. After releasing them for the next 9 h, cells were exposed to thymidine (2 mm) a second time for 15 h. Afterward, cells were released into S-phase for 4 h, and then nocodazole (330 nm) was added to the media for the next 3–4 h to enrich for the mitotic cell population. Kinase activity inhibitors, BI 2536 (500 nm; Calbiochem), hesperadin (500 nm; Calbiochem), reversine (500 nm; Calbiochem), or BAY-320 (10 μm; kindly received from Dr. Gerhard Siemeister, Bayer GmbH, Berlin) were added in the presence of the proteasome inhibitor, MG132 (10 μm; Calbiochem), to the cells for 90 min before fixing these cells for immunofluorescence.

### Chromosome alignment

For analysis of the effect of CENP-F depletion on chromosome alignment, cells were fixed after RNAi either asynchronously or after an additional treatment with 10 μm MG-132 for 2 h. Cells were stained for CENP-F, tubulin, and CREST. DNA was labeled with DAPI. The number of metaphase cells with aligned chromosomes and with misaligned chromosomes was scored for each condition. At least 595 cells (without synchronization) or 92 cells (with synchronization) were analyzed per condition.

## Author contributions

G. C., K. O., and A. M. conceptualization; G. C., K. O., A. P., P. J. H. i. t. V., and S. M. investigation; G. C. and K. O. visualization; G. C., K. O., A. P., P. J. H. i. t. V., C. K., S. W., and S. M. methodology; G. C. and A. M. writing-original draft; G. C., K. O., P. J. H. i. t. V., and S. M. writing-review and editing; A. M. supervision; A. M. funding acquisition; A. M. project administration.

## Supplementary Material

Supporting Information

## Footnotes

4 G. Ciossani and A. Musacchio, unpublished results.

## Footnotes

3 The abbreviations used are:

CENP: centromere protein
CCAN: constitutive centromere associated network
Y2H: yeast two-hybrid
RZZ: Rod/Zwilch/ZW10
SAC: spindle assembly checkpoint
DMEM: Dulbecco's modified Eagle's medium
FBS: fetal bovine serum
TCEP: tris(2-carboxyethyl)phosphine
SEC: size-exclusion chromatography
AUC: analytical ultracentrifugation
MCC: mitotic checkpoint complex
IRES: internal ribosome entry site.
