# Supplementary material for: The kinetochore proteins CENP-E and CENP-F directly and specifically interact with distinct BUB mitotic checkpoint Ser/Thr kinases
Source: J Biol Chem. 2018 May 10;293(26):10084–101. doi: 10.1074/jbc.RA118.003154 (PMC6028960; doi:10.1074/jbc.RA118.003154)
Supplement: Supporting Information [file supp_293_26_10084__index.html]

The kinetochore proteins CENP-E and CENP-F directly and specifically interact with distinct BUB mitotic checkpoint Ser/Thr kinases — Mechanism of kinetochore recruitment of CENP-F — The kinetochore proteins CENP-E and CENP-F directly and specifically interact with distinct BUB mitotic checkpoint Ser/Thr kinases — Mechanism of kinetochore recruitment of CENP-F — Supporting Information 

# The kinetochore proteins CENP-E and CENP-F directly and specifically interact with distinct BUB mitotic checkpoint Ser/Thr kinases

## Supporting Information

- Supplemental Figures - It contains Supplemental Figures and Figure Legends in a single file
