# Supplementary material for: The kinetochore proteins CENP-E and CENP-F directly and specifically interact with distinct BUB mitotic checkpoint Ser/Thr kinases
Source: J Biol Chem. 2018 May 10;293(26):10084–101. doi: 10.1074/jbc.RA118.003154 (PMC6028960; doi:10.1074/jbc.RA118.003154)
Supplement: Supporting Information [file supp_RA118.003154_137019_1_supp_126846_p8d0kq.pdf]

Supplemental Figure Legends

Figure S1. Zwilch and Mad1 localization through the cell cycle

A, **B**) Representative images of Hela cells showing Zwilch (A) and Mad1 (B) localization in the different phases of the cell cycle. Scale bar: 10 µm.

Figure S2. Efficient siRNA depletion of CENP-E, CENP-F and Zwilch

A) Western blot showing CENP-E protein levels in mock treated or CENP-E depleted cells. **B**) Representative images of HeLa cells, either mock-treated or depleted of CENP-E, showing that CENP-E can be efficiently depleted. Scale bar: 10 µm. **C**) Quantification of CENP-E kinetochore levels in cells treated as in panel (B). The graph shows mean intensity of two independent experiments; the error bars indicate SEM and the mean value for non-depleted cells is set to 1. **D**) Western blot showing CENP-F protein levels in mock treated or CENP-F depleted cells. **E**) Representative images of HeLa cells mock treated or depleted of CENP-F showing successful CENP-F depletion. Scale bar: 10 µm. All elements in panel E have already been shown in Figure 2G, and are reproduced here together with the quantification of CENP-F depletion. **F**) Quantification of CENP-F kinetochore levels in cells treated as in panel (E). The graph shows mean intensity of three independent experiments; the error bars indicate SEM and the mean value for non-depleted cells is set to 1. **G**) Quantification of Zwilch kinetochore levels in cells treated as in Figure 2A (*upper panel*) and in (H). The graphs show mean intensity of three (CENP-E experiment) or two (Mad1 experiment) independent experiments; the error bars indicate SEM and the mean value for non-depleted cells is set to 1. **H**) Representative images of HeLa cells mock treated or depleted of Zwilch showing that Zwilch depletion leads to reduced Mad1 levels. Scale bar: 10 µm. **I**) Quantification of Mad1 kinetochore levels in cells treated as in (H). The graph shows mean intensity of two independent experiments; the error bars indicate SEM and the mean value for non-depleted cells is set to 1. **J**) Correlation of Mad1 and Zwilch levels in 38 cells, 19 of which were mock treated, while the other 19 were RNAi depleted of Zwilch. Cells are from two independent experiments. AU, arbitrary units.

Figure S3. Zwilch and BUBR1 co-depletion does not affect CENP-E kinetochore recruitment

A) *Upper panel*: Representative images of HeLa cells either mock treated or co-depleted of CENP-E and CENP-F showing effective co-depletion of the proteins. Scale bar: 10 µm. *Lower panel*: Quantification of co-depletion efficiency. The graph shows mean intensity of two independent experiments; the error bars indicate SEM and the mean values for non-depleted cells are set to 1. **B**) Representative images of HeLa cells mock treated or co-depleted of Zwilch and BUBR1 showing efficient depletion of both the proteins. Scale bar: 10 µm. **C**) Representative images of HeLa cells mock treated or co-depleted of Zwilch and BUBR1 showing that CENP-E localization is not affected. Scale bar: 10 µm. Elements in the left column are negative controls of the RNAi experiment already shown in Figure 2A. **D**) Representative images of HeLa cells mock treated or co-depleted of Zwilch and BUB1 showing efficient depletion of both proteins. Two cells with different Zwilch levels and the same low BUB1 levels are shown for the RNAi condition. Scale bar: 10 µm. **E**) Representative images of Hela cells mock treated or co-depleted of Zwilch and BUB1, showing that CENP-E is not lost from KTs upon Zwilch and BUB1 co-depletion. Two cells with different depletion efficiency for Zwilch are shown for the RNAi condition. Scale bar: 10 µm.

Figure S4. Kinetochore localization of RZZ (Zwilch) is not affected by CENP-E or CENP-F depletion also in presence of microtubules

A-**C**) Representative images of a prometaphase and metaphase HeLa cells either mock treated (A) or individually depleted of CENP-F (B), co-depleted of both CENP-E and CENP-F (C), individually depleted of CENP-E (D) in absence of nocodazole. Neither CENP-E nor CENP-F are required for kinetochore localization of Zwilch even in the presence of spindle microtubules. Scale bar: 10 μm.

Figure S5. Mitotic phenotype of CENP-F depletion

A) Examples of the scored categories, aligned metaphase and metaphases with misalignments in mock treated or CENP-F depleted HeLa cells. Scale bar: 10 μm. **B**) Percentages of cells in the depicted categories in unsynchronized cells from one experiment. **C**) Percentages of cells in the depicted categories in cells treated with 10 μM MG for 2h before fixation from one experiment. **D**) Mean duration of mitosis of HeLa cells in presence or absence of endogenous CENP-F. Cell morphology was used to measure entry into and exit from mitosis by time-lapse microscopy (n>76 per condition per experiment) from two independent experiments. Error bars indicate SEM.

Figure S6. CENP-E kinetochore localization sensitivity to kinases inhibition

A-**D**) Representative images and quantification of CENP-E kinetochore levels in mitotic Hela cells treated with the indicated concentrations of the indicated kinase inhibitors. Scale bar: 10 μm. CENP-E localization is sensitive to Aurora B (A), Mps1 (B) and, to a lesser extent, Plk1 (D) but not to BUB1 (C) inhibition. Reduction in P-T232 Aurora B (Aurora B activation segment) staining was used as a positive control for Aurora B inhibition (A), while reduction in BUB1 localization was used as positive control for Mps1 inhibition (B). BUB1 inhibition was confirmed by reduction in P-T121 H2A staining (C). The graphs show mean intensity of one (C, I), two (A) or four (B) experiments. The error bars indicate SEM and the mean values for DMSO-treated cells are set to 1.

Figure S7. Sensitivity to kinases inhibition of CENP-F kinetochore localization

A-**E**) Representative images and quantification of CENP-F kinetochore levels in mitotic Hela cells treated with the indicated type and concentrations of kinase inhibitors. Scale bar: 10 μm. CENP-F localization is sensitive to Mps1 (A) and partially to Plk1 (D) inhibition. Despite a requirement for the BUB1 kinase domain for CENP-F kinetochore recruitment, BUB1 catalytic activity is dispensable (B, C). Reduction in BUB1 localization was used as a control for Mps1 inhibition (A) and reduction in P-T121-H2A staining was used as a control for BUB1 inhibition (E). Loss of Plk1 localization was used as control for Plk1 inhibition (D). The graphs show mean intensity of one (B, C, E) or two (A, D) experiments. The error bars indicate SEM and the mean values for DMSO-treated cells are set to 1. **F**) Representative images of HeLa cells mock treated or depleted of CENP-F showing that CENP-F depletion does not affect the kinase activity of BUB1, as no changes are detected for the P-T121-H2A signal. Scale bar: 10 μm.

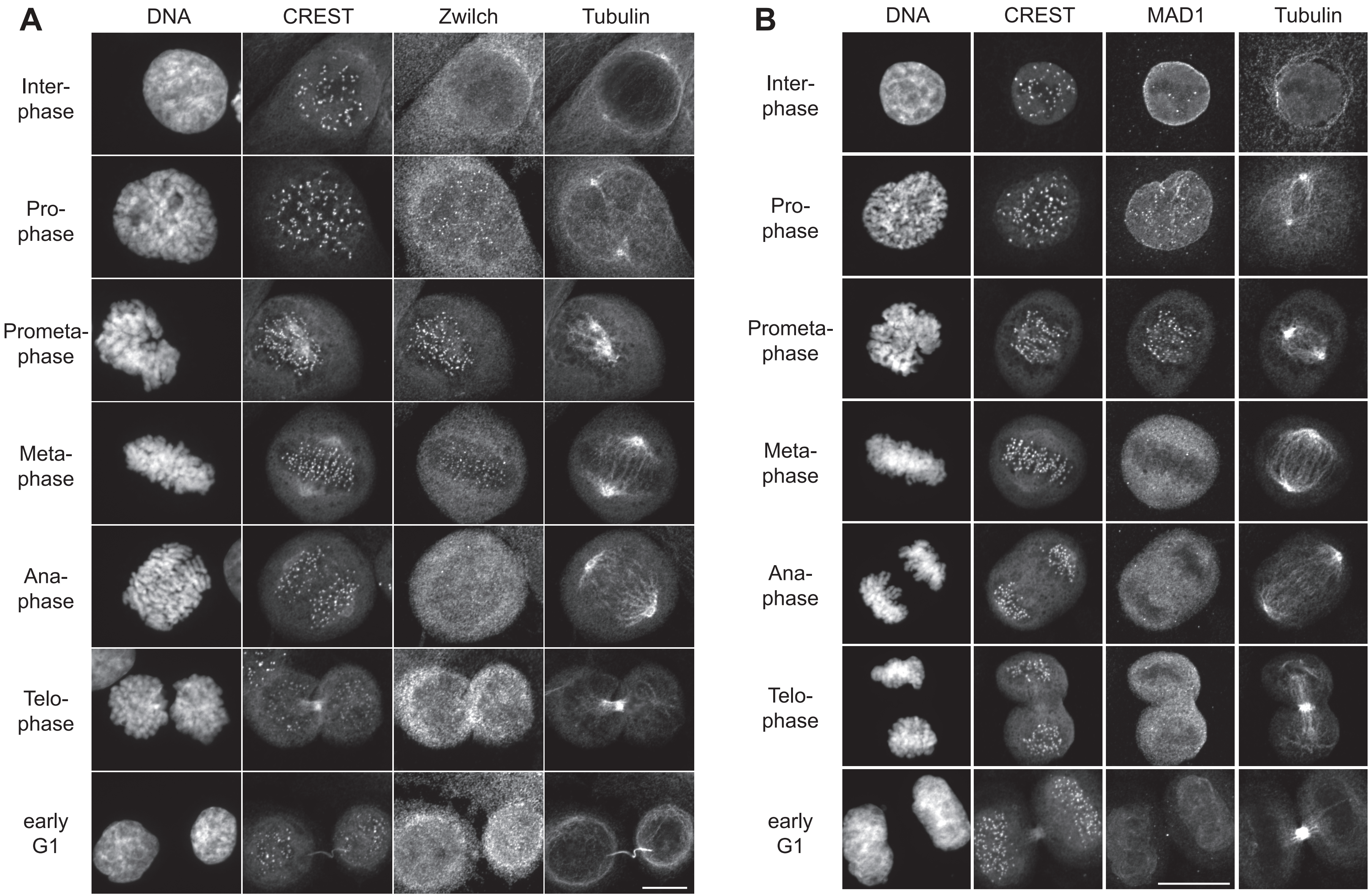

Ciossani, Overlack et al. Figure S1

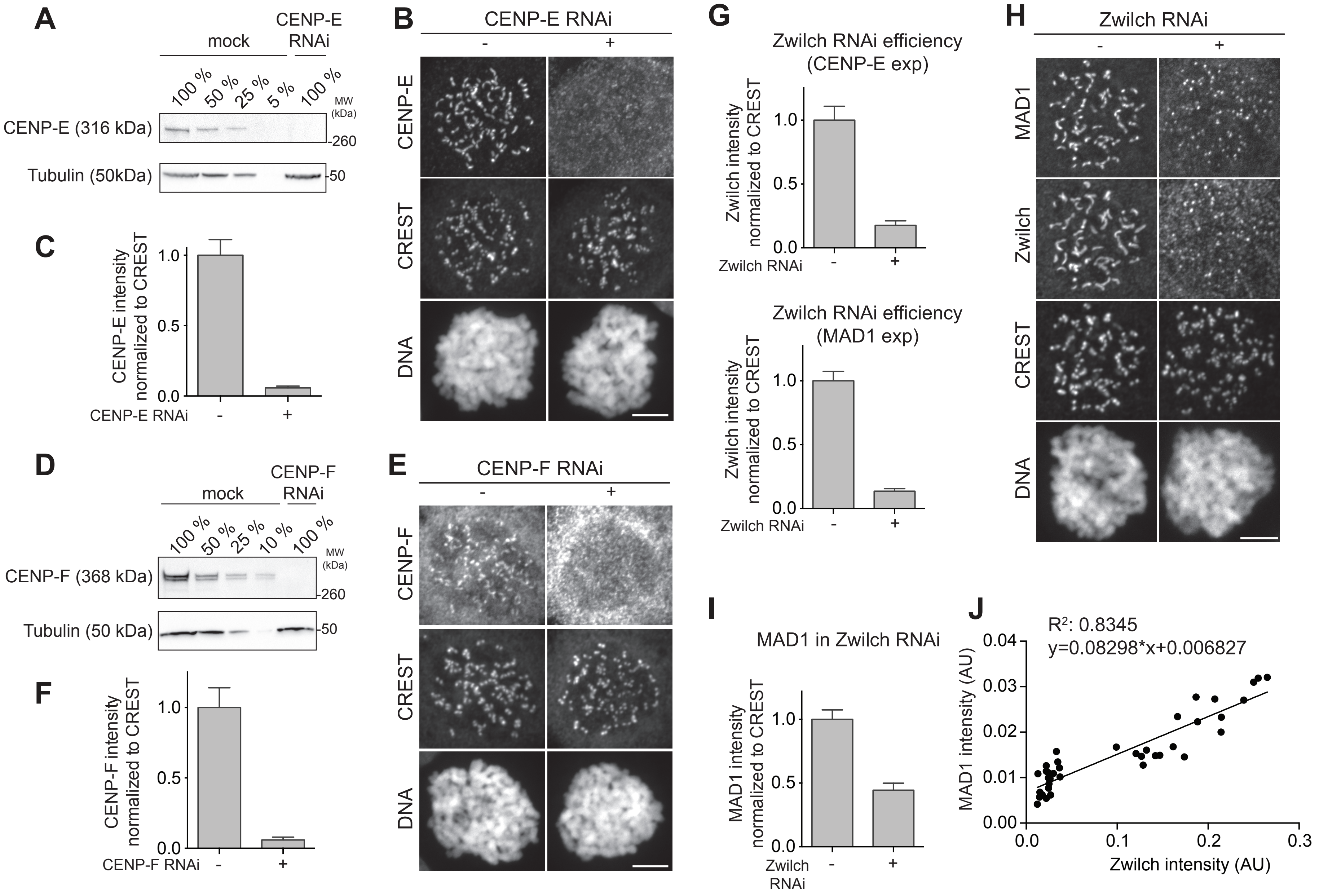

Ciossani, Overlack et al. Figure S2

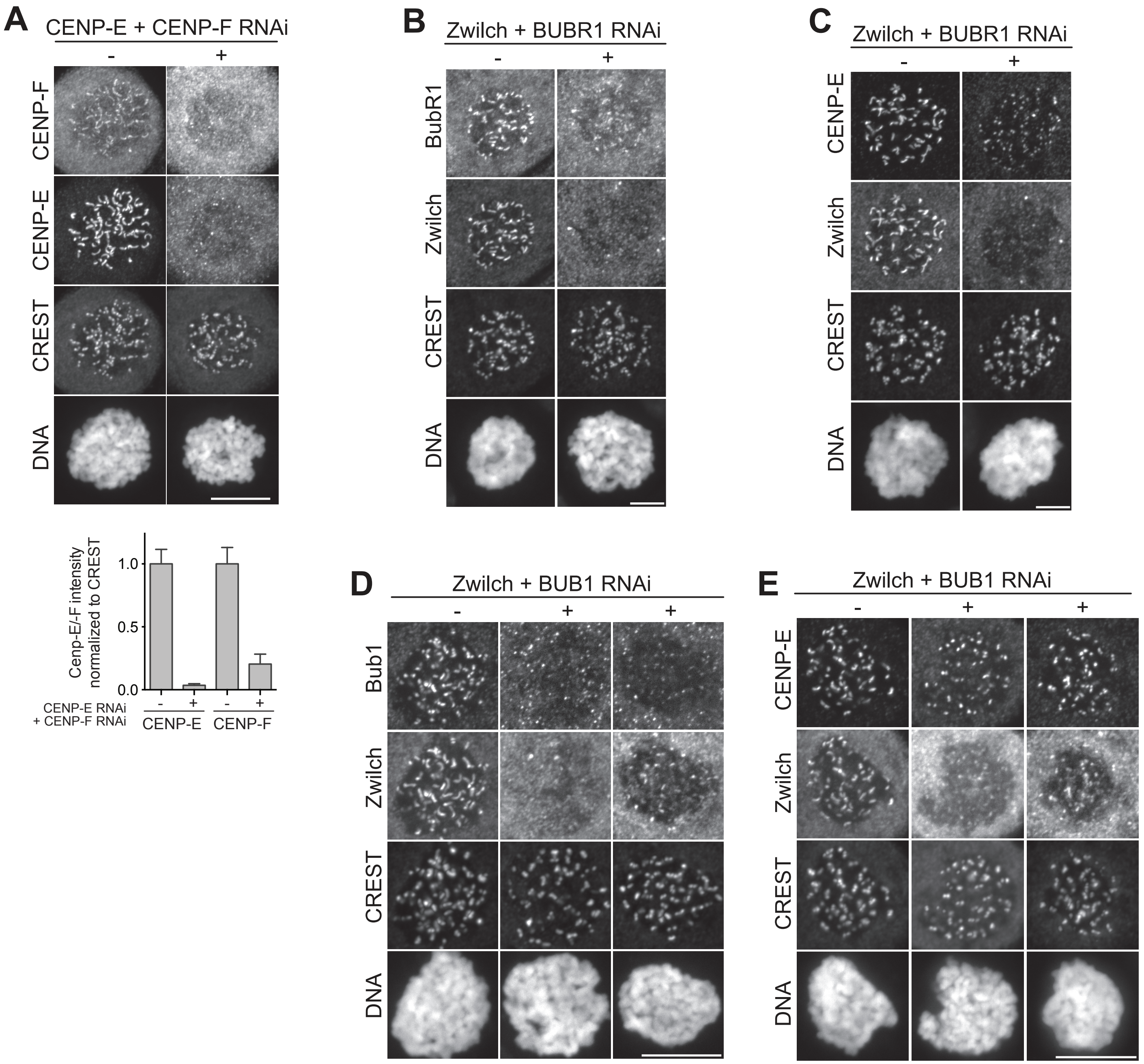

Ciossani, Overlack et al. Figure S3

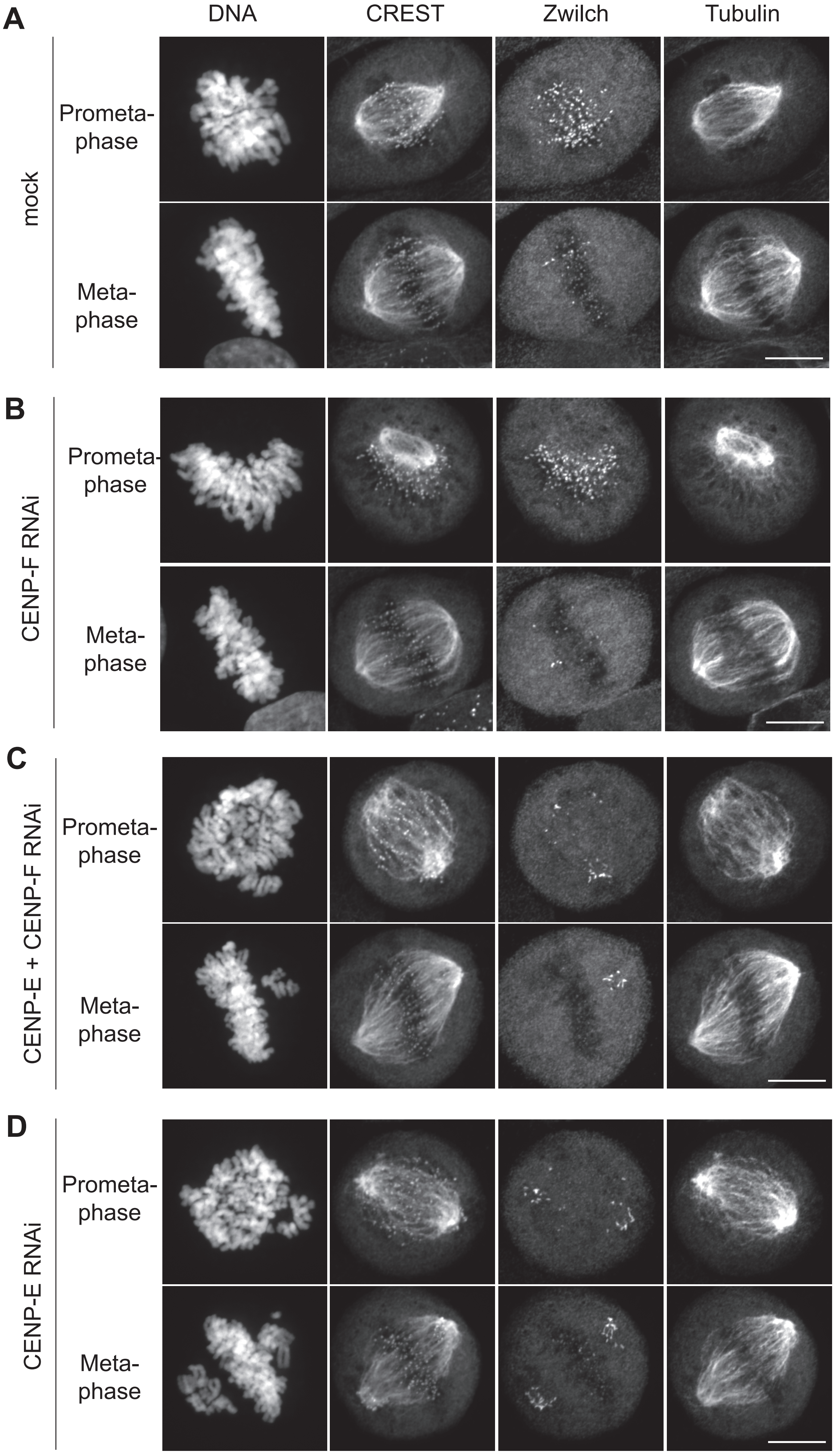

Ciossani, Overlack et al. Figure S4

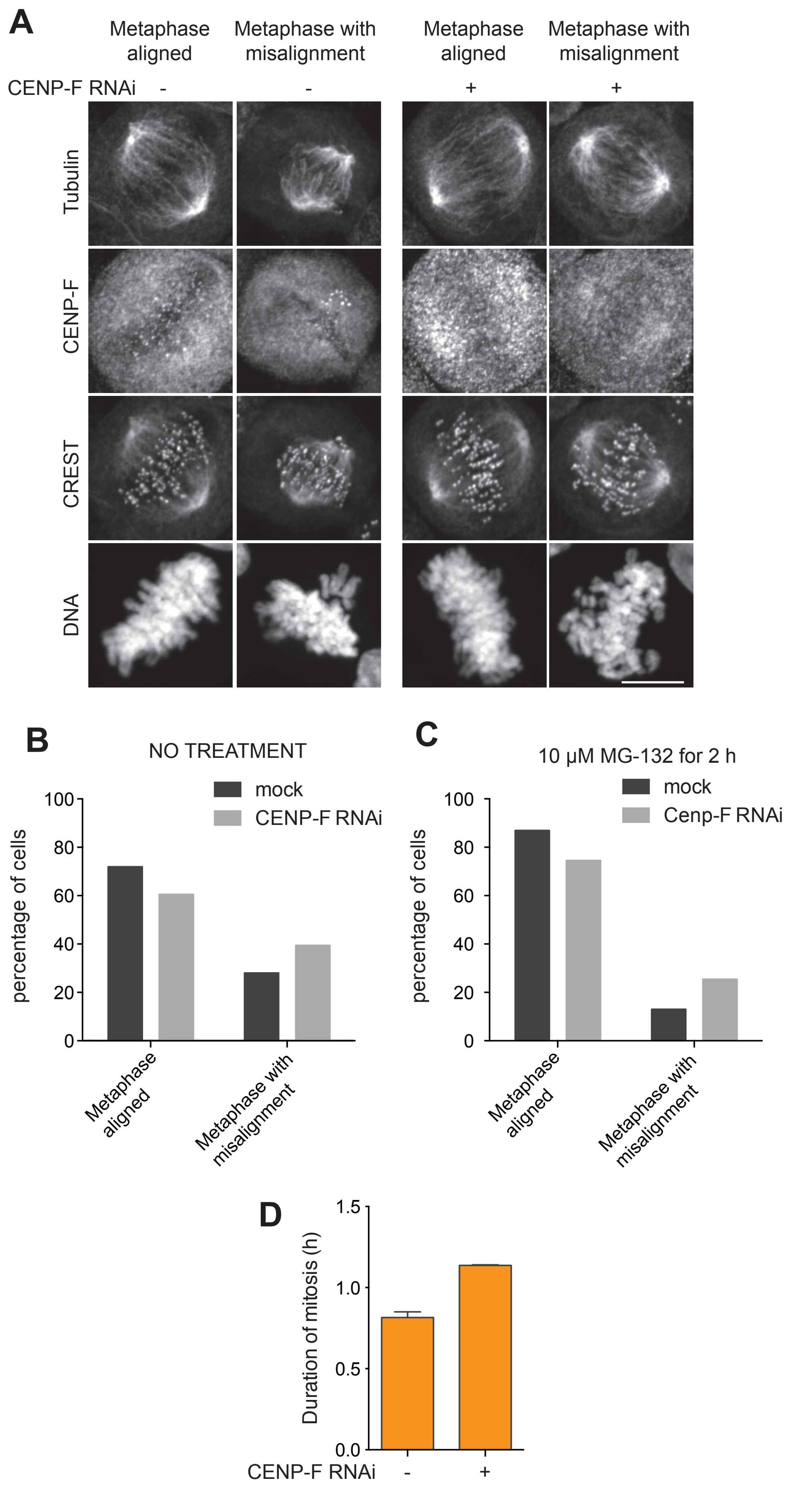

Ciossani, Overlack et al. Figure S5

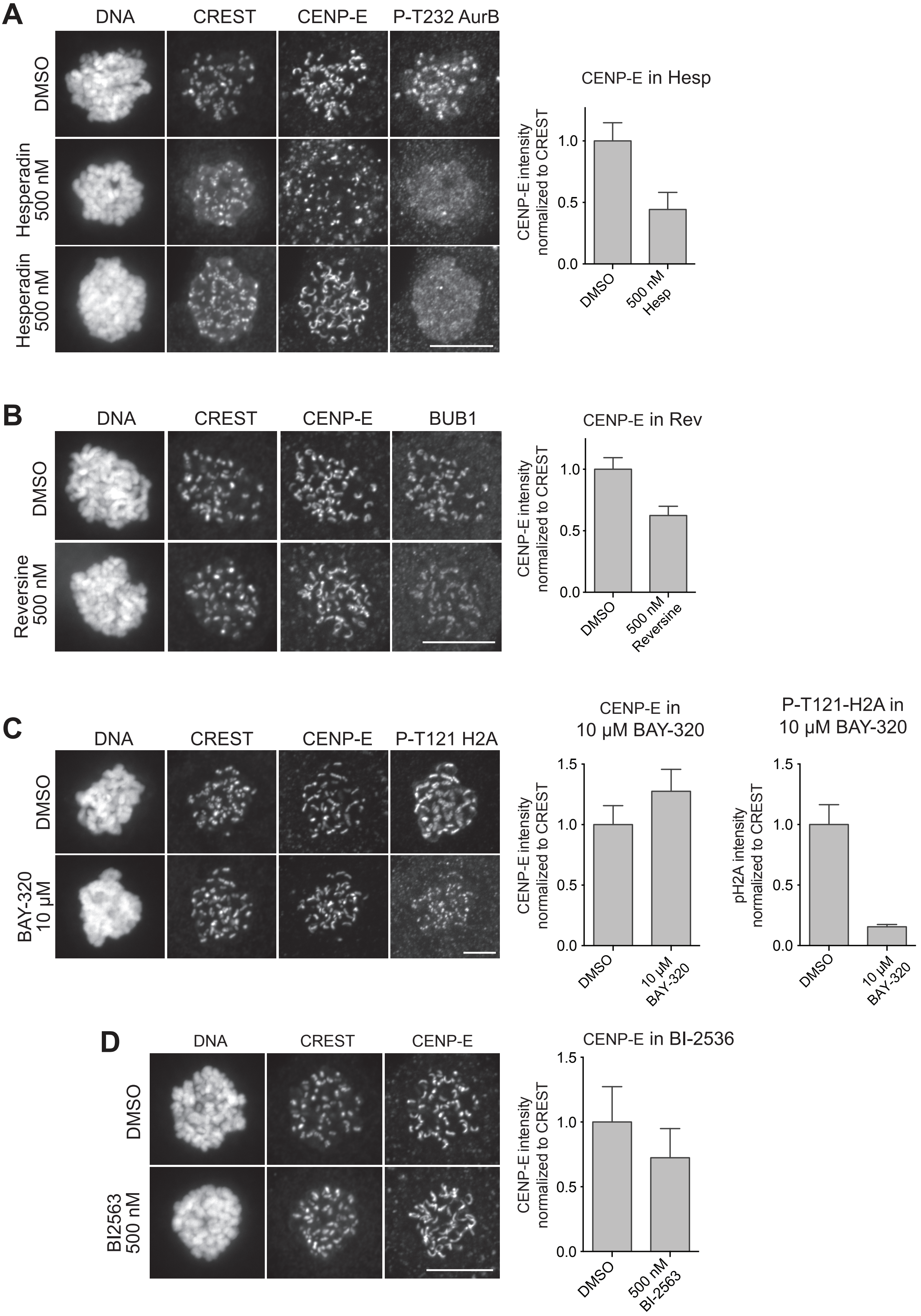

Ciossani, Overlack et al. Figure S6

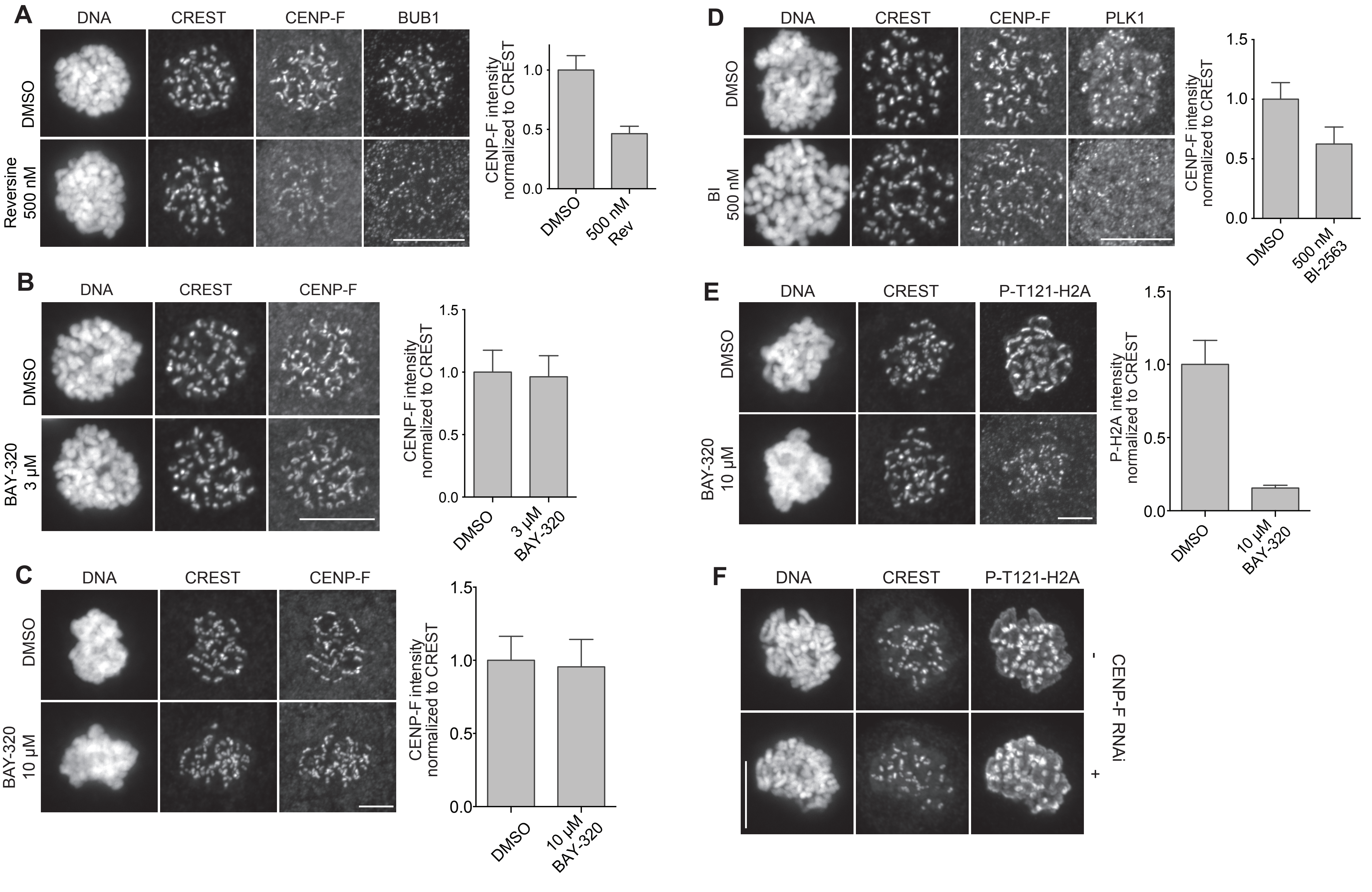

Ciossani, Overlack et al. Figure S7
